# The bacterial Sec system is required for the organization and function of the MreB cytoskeleton

**DOI:** 10.1371/journal.pgen.1007017

**Published:** 2017-09-25

**Authors:** Sutharsan Govindarajan, Orna Amster-Choder

**Affiliations:** Department of Microbiology and Molecular Genetics, IMRIC, The Hebrew University Faculty of Medicine, Jerusalem, Israel; Universidad de Sevilla, SPAIN

## Abstract

The Sec system is responsible for protein insertion, translocation and secretion across membranes in all cells. The bacterial actin homolog MreB controls various processes, including cell wall synthesis, membrane organization and polarity establishment. Here we show that the two systems genetically interact and that components of the Sec system, especially the SecA motor protein, are essential for spatiotemporal organization of MreB in *E*. *coli*, as evidenced by the accumulation of MreB at irregular sites in Sec-impaired cells. MreB mislocalization in SecA-defective cells significantly affects MreB-coordinated processes, such as cell wall synthesis, and induce formation of membrane invaginations enriched in high fluidity domains. Additionally, MreB is not recruited to the FtsZ ring in *secA* mutant cells, contributing to division arrest and cell filamentation. Our results show that all these faults are due to improper targeting of MreB to the membrane in the absence of SecA. Thus, when we reroute RodZ, MreB membrane-anchor, by fusing it to a SecA-independent integral membrane protein and overproducing it, MreB localization is restored and the defect in cell division is corrected. Notably, the RodZ moiety is not properly inserted into the membrane, strongly suggesting that it only serves as a bait for placing MreB around the cell circumference. Finally, we show that MreB localization depends on SecA also in *C*. *crescentus*, suggesting that regulation of MreB by the Sec system is conserved in bacteria. Taken together, our data reveal that the secretion system plays an important role in determining the organization and functioning of the cytoskeletal system in bacteria.

## Author summary

The notion that bacterial cells have intricate spatial organization, which affects many vital processes, is relatively new and, hence, the underlying mechanisms are largely unknown. The general secretion system and the cytoskeleton are central systems, each known to organize functions associated with certain cellular domains, in both eukaryotes and prokaryotes. While the role of the Sec system in membrane protein translocation and secretion has been largely explored, not much in known about its role in inner cell organization. We show that the Sec system is important for the localization pattern and functionality of the bacterial cytoskeletal system, which controls cell shape, cell division and polarity. Our findings highlight the Sec system as a central coordinator that controls cellular functions on both sides of the membrane.

## Introduction

Internal organization of bacterial cells is a complex process coordinated spatiotemporally by several molecular machineries. In most rod-shaped bacteria, the actin-homolog, MreB, functions as an intracellular organizer controlling cell wall synthesis, cell shape maintenance, cell polarity, cell division and more [1,2,3]. Consequently, disruption of the MreB cytoskeletal system leads to pleiotropic phenotypes, including disorganized cell wall synthesis, loss of rod shape, mislocalization of proteins and aberrant chromosome organization [2]. The subcellular organization of the MreB filaments themselves has been at the center of an ongoing debate. Initially, MreB was suggested to form continuous helical filaments along the long axis of the cell underneath the cell surface [4]. However, later high-resolution studies suggested that MreB forms discrete, short patches of filaments that move perpendicular to the long axis of the cell via force generated by cell wall synthesis [5,6,7]. Then again, more recent reports documented the existence of extended MreB filaments, which are sometimes helical [8], leaving the issue of the exact subcellular organization of MreB still in doubt [1].

Dynamic physical interaction between MreB and cell wall biosynthetic enzymes support a model by which MreB serves as a scaffold for cell wall synthesis [9], whereas the process of cell wall synthesis drives MreB circumferential motions [5,6,7]. Recently, RodZ was also demonstrated to participate in MreB rotation by assisting in coupling it to the cell wall biosynthetic enzymes [10]. In addition to its role in lateral cell wall synthesis and cell elongation, MreB is also implicated in septal cell wall synthesis through its interaction with FtsZ, the bacterial tubulin homolog [11,12]. Thus, as a result of MreB-FtsZ interaction, penicillin-binding proteins (PBPs), the major peptidoglycan (PG) synthetic enzymes that are bound to MreB, were suggested to be recruited to the septum without the requirement for other cell division proteins [11,12]. Thus, the MreB cytoskeletal system and the cell wall synthetic machinery are interlinked and disruption of either of these systems affects the organization and function of the other.

MreB filaments are positioned at the inner surface of the cytoplasmic membrane, whereas MreC and MreD, its cytoskeletal partners, are integral membrane proteins, with MreC having a large periplasmic domain and MreD being largely membrane-embedded [13]. Association of MreB with the membrane requires its membrane insertion loop and, in some bacteria, it is further assisted by an N-terminal amphipathic helix [14]. Recent studies indicated that lipid-linked PG precursors are also important for the association of MreB with the membrane [15]. *In vivo* observations using specific lipid-binding dyes showed that the assembly of MreB filaments with the membrane generates fluid lipid domains and promotes movement of membrane proteins and lipids [16], similar to actin cortical cytoskeleton of eukaryotes [17]. While the association of MreB with the cell membrane has been broadly studied [14,15,18], the possible involvement of membrane-organizing systems in MreB localization and function is largely unexplored.

The Sec protein translocation pathway is involved in biogenesis of a large number of membrane-bound and secreted proteins in most bacteria (reviewed in [19] and [20]). The Sec system is comprised of the membrane-embedded SecYEG translocon, which forms the pore through which polypeptides are translocated in unfolded conformation [21], the SecA ATPase, which functions as the motor protein driving protein translocation [22] and the SecB chaperone, which maintains the newly synthesized proteins in an unfolded conformation[23]. Depending on the type of protein cargo that needs to be transported, the Sec system also cooperates with the Signal Recognition Particle (SRP) pathway [24]. The substrates of the Sec system generally encompass an N-terminal signal sequence, which gets proteolytically cleaved by the signal peptidase during translocation [25]. The Sec system has been extensively studied for its role in membrane protein targeting and secretion, with few studies suggesting that it is involved in targeting membrane or secreted proteins specifically to the poles [26,27].

Although MreB is not an integral membrane protein and does not have a Sec-type signal sequence, three types of data encouraged us to investigate the relationship between the main bacterial membrane translocation machinery and the MreB cytoskeleton. First, a high-throughput survey of protein interactions in *E*. *coli* suggested that SecA and MreB are interaction partners [28]. Second, in *E*. *coli* cells depleted for SecE, MreB was found to be enriched in the cytoplasm [29]. Finally, in yeast cells, disruption of the Sec system was shown to affect organization of the MreB-structural homolog, actin [30].

Here we show that SecA and MreB interact genetically and that the organization and function of MreB is regulated by the Sec system. Upon inactivation or depletion of components of the Sec machinery, in particular SecA, MreB changes its localization pattern and accumulates mainly at polar or sub-polar sites. MreB mislocalization in *secA* mutant cells results in disordered cell wall formation and generation of multilayer membrane regions, which are enriched with high fluidity domains. Furthermore, the mislocalized MreB in *secA* mutant cells is not efficiently recruited to the Z-ring resulting in incomplete cell division and filamentation. We demonstrate that the above defects are due to RodZ, MreB partner protein, not getting to the membrane in the absence of active SecA, because when we reroute and overexpress RodZ through a SecA-independent pathway, MreB localization and the division defect of SecA-inactivated cells are partially corrected. Finally, we show that the SecA-dependent mechanism for MreB localization exists also in *Caulobacter crescentus*, suggesting that it is conserved across diverged gram negative bacteria. Taken together, our findings underscore a previously unrecognized capability of the membrane translocation system in organizing bacterial cells on both sides of the membrane.

## Results

### *secA* and *mreB* genetically interact

**Fig 1:**
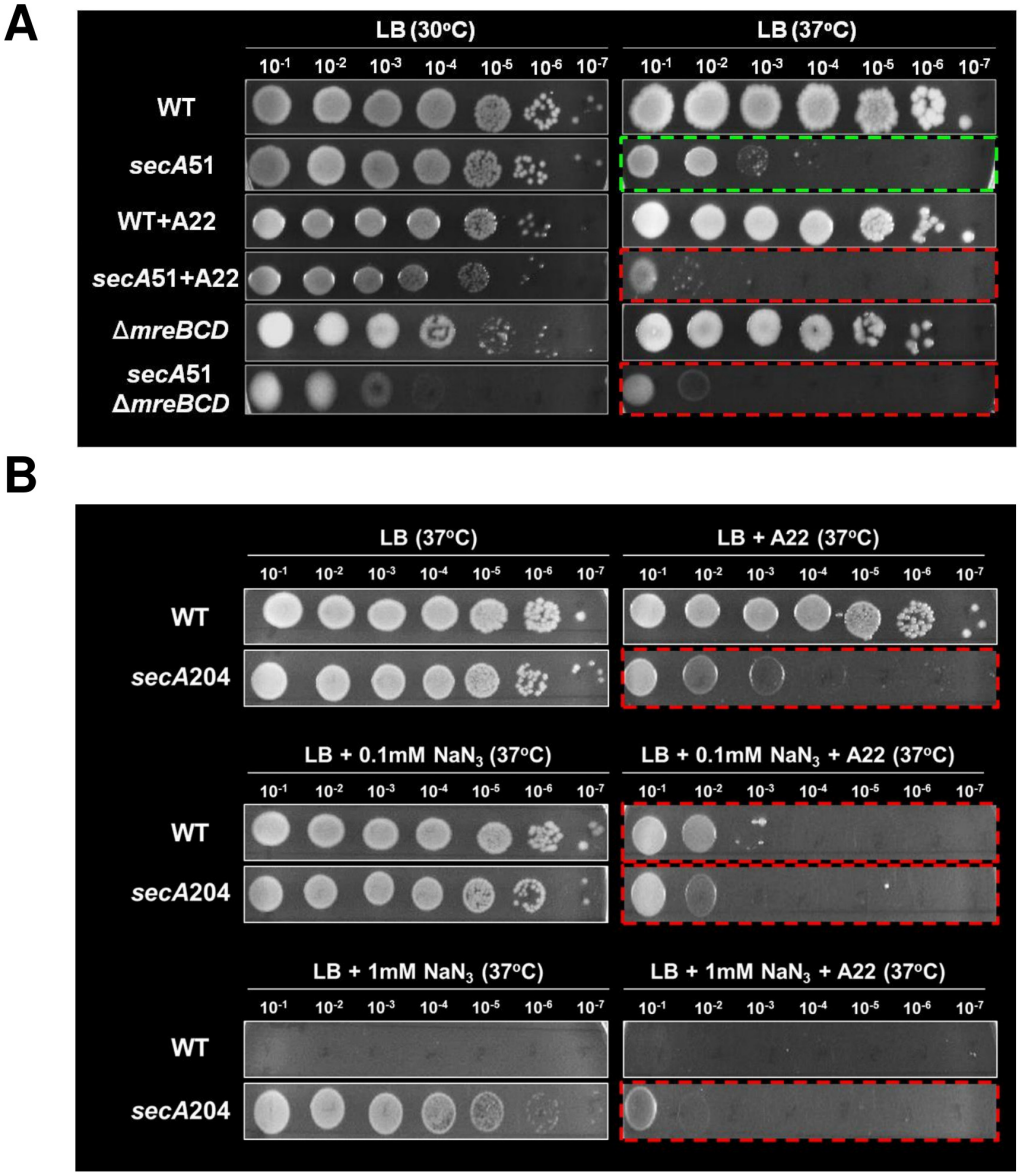
MreB and SecA interact genetically. (**A**) Pictures of wild-type (MG1655) and *secA*51 cells spotted after serial dilutions on LB plates with or without sub-inhibitory concentration of A22 (1 μg/ml) and grown for 20 hours at the permissive (30°C) or semi-permissive (37°C) temperature. Also shown are LB plates with serial dilutions of Δ*mreBCD* and *secA*51Δ*mreBCD* mutant cells grown for 20 hours at the permissive (30°C) or semi-permissive (37°C) temperature. (**B**) Pictures of wild-type (MC4100) and *secA*204 (sodium azide resistant) cells spotted after serial dilutions on LB plates with or without sub-inhibitory concentration of A22 (1 μg/ml) and with (0.1 mM or 1 mM) or without sodium azide. All the plates were grown for overnight at 37°C.

In order to test if the Sec system and MreB cooperate with each other, we first asked whether the genes encoding the Sec proteins and MreB interact genetically. For this purpose, we compared the growth of cells defective in either the *sec* genes or *mreB* to that of cells defective in both. We initiated this survey by spotting serial dilutions of wild-type *E*. *coli* and *secA*51 mutant, which carries a temperature sensitive mutation that renders the cell highly defective in Sec functions [31], on LB agar plates containing or not containing a sub-inhibitory concentration of A22, which inhibits MreB polymerization by directly binding to its ATP-binding domain [32]. In addition, we also spotted serial dilutions of Δ*mreBCD* cells, which lack the entire MreBCD complex, (enabled by the presence of suppressor mutations, see [33]) and of *secA*51Δ*mreBCD* double mutant cells, which are defective in both systems, on LB agar plates. The plates were incubated at the permissive temperature (30°C) or at a semi-restrictive temperature (37°C). The results, compiled in Fig 1A, show that already at the permissive temperature, complete depletion of MreB from *secA*51 cells via a deletion (the double mutant) attenuated cell growth, as evidenced by reduction in colony forming ability, raising the possibility of synthetic inhibition upon manipulation of the two genes (treatment by a sub-inhibitory concentration of A22 had only a minor effect). The results at the semi-restrictive temperature confirmed this possibility, since both cultures of *secA*51, in which MreB had been either inhibited by A22 or absent due to a deletion, exhibited at least 20 fold reduction in their colony forming ability compared to *secA*51 cells in which the MreB level or activity have not been manipulated (Fig 1A, compare growth in the two boxes bordered by red dashed lines to growth in the box bordered by a green dashed line). The reduction in the ability to form colonies is more likely to be a synthetic effect, rather than an additive effect of two mutations, since treatment with sub-inhibitory concentration of A22 specifically affected the *secA*51 cells and not the wild-type.

Next, we compared the sensitivity of wild-type cells and of *secA*204 cells, which carry the *prlD*21 mutation (*prlD* is the former name of *secA*) that renders them resistant to sodium azide (NaN_3_), to the MreB inhibitor A22. To this end, we spotted serial dilutions of the two strains on LB agar plates lacking or containing 0.1 mM NaN_3_ or 1 mM NaN_3_, supplemented or not with a sub-inhibitory concentration of A22, and incubated the plates overnight at 37°C. The results, compiled in Fig 1B, show that both strains grew similarly on LB agar lacking or containing low concentration of NaN_3_ (0.1 mM), whereas only *secA*204 cells, which are resistant to sodium azide, formed colonies on LB agar containing 1 mM NaN_3_, as expected (Fig 1B, left panel). When A22 was included in the plate, growth of wild-type cells was not affected in the absence of NaN_3_, but was severely inhibited in the presence of only 0.1 mM NaN_3_. In contrast, growth of *secA*204 cells was drastically inhibited in the presence of A22, and the inhibition became even more severe in the presence of NaN_3_ (Fig 1B, right panel). Since the *secA*204 cells are known to be defective in secretion, apparently because the mutation slows the ATPase activity of SecA [34], it is most likely that their A22 hypersensitivity phenotype of is due to the secretion defect. The possibility of ineffective efflux system contributing to the increased sensitivity of SecA-defective cells to A22 cannot be completely ruled out. However, our observation that the *secA*51Δ*mreBCD* double mutant cells exhibit a pronounced growth defect at the semi-restrictive temperature suggests that A22 hypersensitivity of *secA*51 cells is less likely to be caused by an inefficient efflux system.

Finally, we tested whether mutations in other *sec* genes, namely *secE*15 and *secY*39, both of which defective in secretion at low temperatures, also exhibit growth defect in the presence of a sub-inhibitory concentration of A22. To test this, we spotted serial dilutions of overnight-grown cultures of wild-type, *secE*15 and *secY*39 on LB agar plates, supplemented or not with A22, and incubated them at the permissive (37°C), semi-restrictive (30°C) or restrictive (23°C) temperatures. The results, compiled in S1 Fig, show that, unlike SecA-defective cells, SecE- or SecY-defective cells did not show altered sensitivity to A22 at the permissive (37°C) or semi-restrictive (30°C) temperatures, while their growth was completely inhibited at the restrictive temperature (23°C).

Taken together, the data presented above shows that co-inhibition of SecA and MreB largely compromise cell growth, compared to inhibition of only one of them, strongly suggesting that the genes encoding them interact genetically.

### SecA is important for subcellular localization of the MreB cytoskeleton

**Fig 2:**
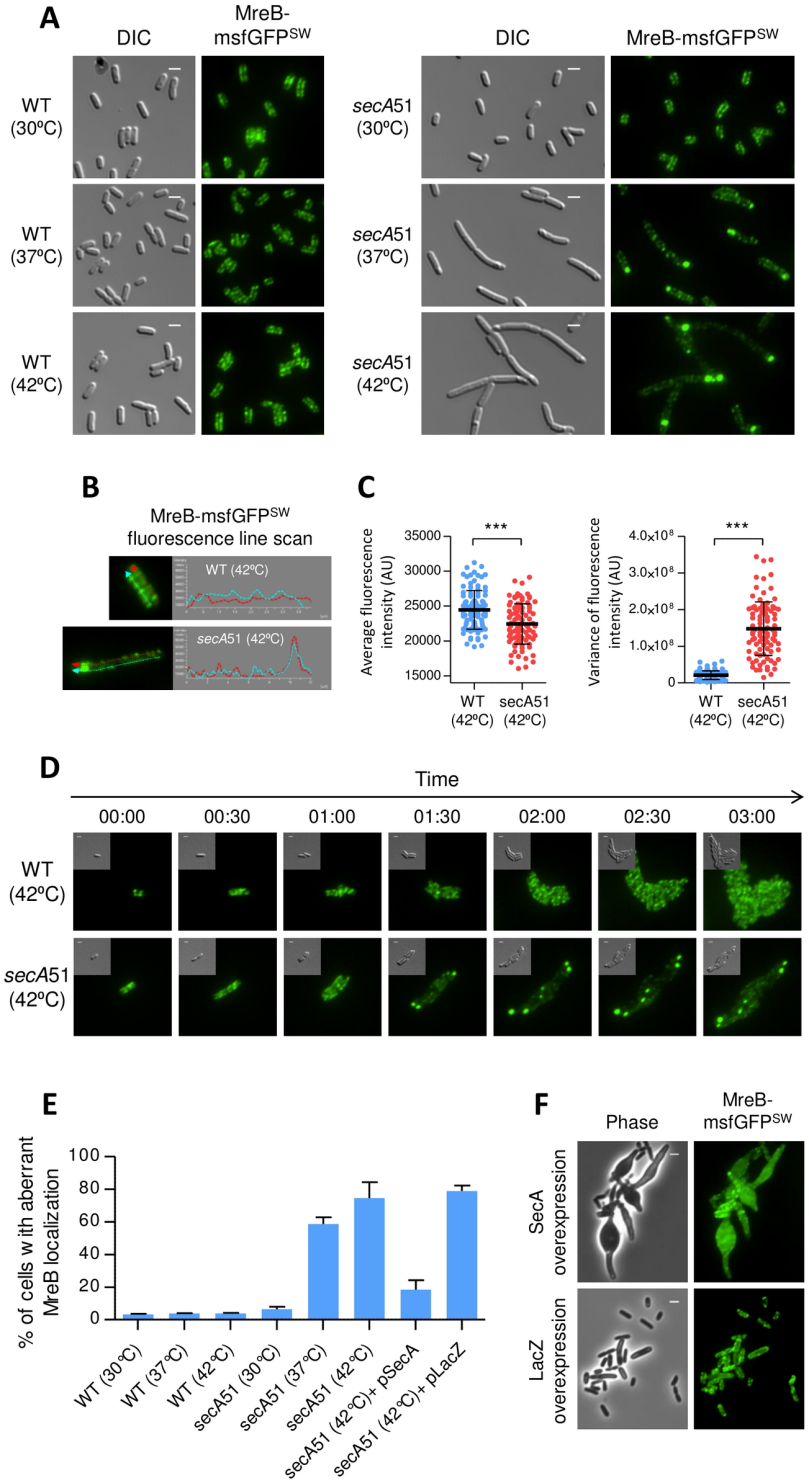
SecA affect localization of the MreB cytoskeleton. **(A)** Images of wild-type and *secA*51 mutant cell, which express MreB-msfGFP^SW^, grown at the permissive (30°C), semi-restrictive (37°C), or restrictive (42°C) temperature. (**B**) Line scan analysis showing the distribution of MreB-msfGFP^SW^ on both sides of the membrane along the cell length of wild-type and *secA*51 mutant. The red and cyan arrowed lines correspond to traces that were scanned to determine the fluorescence intensity. Fluorescence intensities (AU) are presented as plots next to each panel. (**C**) Distribution of average fluorescence intensity (left) and variance of fluorescence intensity (right) of wild-type and *secA*51 mutant cells grown at the restrictive (42°C) temperature. Values were obtained from nearly 100 cells from two independent experiments. The black bars represents the mean value ± standard deviation of more than 90 cells from each sample obtained from two independent experiments. The statistical significance was calculated using unpaired t-test analysis (* p<0.05; *** p<0.0001). **(D)** Time-lapse microscopy images of wild-type (upper panels) and *secA*51 mutant (lower panels) cells producing MreB-msfGFP^SW^ and grown at the non-permissive temperature (42°C). **(E)** Percentage of cells with mislocalized MreB. In all cases, aberrant MreB localization refers to cells containing mislocalized MreB foci that are positioned at polar, sub-polar or other ectopic regions. More than 500 cells from each sample were analyzed from three independent experiments. Means and standard deviations are shown. **(F)** Images of cells expressing MreB-msfGFP^SW^ from the chromosome and overexpressing wild-type SecA or LacZ from a plasmid. The GFP fusion protein was observed by fluorescence microscopy and cells were observed with DIC or phase microscopy. Shown are DIC (grey), phase (grey) and GFP (green) fluorescence signal. Scale bar corresponds to 2 μm.

Having observed that SecA and MreB interact genetically, we asked whether localization of the two proteins depend upon each other. First, we asked whether SecA affects the subcellular organization of the MreB cytoskeletal protein in *E*. *coli*. For this purpose, we monitored the localization of an MreB tagged with monomeric superfolder green fluorescent protein (MreB-msfGFP^SW^ a sandwich fusion shown to be functional *in vivo* [10]), expressed from its native chromosomal locus, in wild-type and in *secA*51 cells, grown at the permissive (30°C), semi-restrictive (37°C) and restrictive (42°C) temperatures. In wild-type cells, MreB-msfGFP^SW^ was unaffected at all temperatures and was observed as puncta distributed along the cell periphery (Fig 2A, left panels, and Fig 2E). However, whereas the pattern of MreB-msfGFP^SW^ localization in the *secA*51 mutant cells grown at the permissive temperature (30°C) was similar to that observed in wild-type cells, in cells grown at the semi-restrictive (37°C) or the restrictive (42°C) temperatures, MreB-msfGFP^SW^ exhibited an entirely different distribution pattern (Fig 2A, right panels, and Fig 2E). Under these conditions, the *secA*51 cells became elongated, placing a considerable amount of the MreB-msfGFP^SW^ molecules (24 ±7% of total cellular MreB, n = 50 cells) in foci at polar and sub-polar regions (Fig 2A, right panels). Line scan analysis on both sides of the membrane along the cell length indicated that the intensity profile of MreB in *secA*51 and in wild-type cells is clearly different (Fig 2B). Further quantification of the fluorescence intensities revealed that the average fluorescence intensity, which estimates the relative amount of MreB, is somewhat lower in *secA*51 cells compared to the wild-type cells (Fig 2C, left), whereas the variance in fluorescence distribution, which provides information on non-homogeneous distribution of MreB, is much higher in *secA*51 cells compared to wild-type, reflecting the presence of aberrantly distributed MreB clusters in these cells (Fig 2C, right). The observed changes in MreB-msfGFP^SW^ localization are not due to cleavage of the chimeric protein that releases GFP, since Western blot analysis of wild-type and *secA*51 cells expressing MreB-msfGFP^SW^ using α-GFP antibodies revealed that the MreB-msfGFP^SW^ fusion protein is relatively stable and the negligible amount of what might be free GFP is the same in wild-type and *secA*51 cells grown at the restrictive (42°C) temperature (S2 Fig). Notably, we obtained similar results when we used MreB–red fluorescent protein sandwich fusion (MreB-RFP^SW^) [35] as a reporter for MreB localization (S3A Fig).

Next, we followed MreB localization upon SecA inactivation by time-lapse microscopy. When wild-type cells expressing MreB-msfGFP^SW^ were grown at the restrictive temperature, no defect in MreB localization was observed (Fig 2D, upper panels, and S1 Movie). However, two hours after shifting the *secA*51 cells to 42°C, a considerable amount of the MreB-msfGFP^SW^ molecules accumulated in clusters located mainly near the cell poles. As growth of the *secA*51 cells at the non-permissive temperature continued, cells became filamented and the MreB-msfGFP^SW^ continued to concentrate mainly at polar or sub-polar sites (Fig 2D, lower panels, and S2 Movie). Evidently, the *secA*51 cells did not become spherical when grown at the restrictive temperature, probably due to the fraction of MreB molecules that localized along the cell membrane and remained dynamic (S3 Movie). Importantly, mislocalized MreB-msfGFP^SW^ in *secA*51 cells grown at the non-permissive temperature (42°C) could be reversed to localize as in wild-type cells when shifted back to the permissive temperature (30°C) (S3B Fig and S4 Movie). This result indicates that MreB localization is greatly affected by the protein secretion status of the cells and that *secA*51 cells in which MreB is mislocalized are not dead. The ability of MreB to resume its normal localization pattern when shifted back to non-restrictive conditions is not surprising in light of the demonstrated spatial plasticity of MreB. Thus, during the cell cycle, MreB condenses to mid-cell to co-localize with the division plane-associated ring and expands back after cell division [11,12,36]. Still, the possibility that de novo MreB synthesis during the experiment contributes to the reversal cannot be ruled out.

To confirm that the Sec system in the *secA*51 mutant is defective under the conditions used, we tested the localization pattern of super-folder GFP fused to MalE, which is a known substrate of the Sec system and which has been shown to localize to the cell poles [37,38]. The results in S3C Fig demonstrate that polar localization of MalE-sfGFP was abolished in *secA*51 cells, but not in wild-type cells, grown at the restrictive temperature, indicating that the Sec system is indeed impaired in *secA*51 under the conditions used. The wild-type-like localization pattern of MreB-msfGFP^SW^ was restored in *secA*51 cells upon ectopic expression of SecA (S3D Fig, left panels, and Fig 2E), but not upon expression of an unrelated protein, LacZ (S3D Fig, right panels, and Fig 2E). Mislocalization of MreB-RFP^SW^ was also observed when SecA was depleted from DRH729, a strain that expresses *secA* from an inducible promoter (S3E Fig).

In wild-type cells, approximately half of the cellular SecA content is associated with the inner membrane, whereas the other half is soluble [39]. Since SecA is an interaction partner of MreB [28], we hypothesized that when it is overexpressed, the amount of SecA in the cytoplasm will increase, and a significant fraction of it would be available to interact with MreB, keeping it in the cytoplasm. Such a scenario is expected to affect cell shape, since it will reduce the pool of membrane-associated MreB, which is involved in cell wall synthesis. To test this hypothesis, we overexpressed SecA in cells expressing MreB-msfGFP^SW^ as the sole source of MreB. The results, presented in Fig 2F upper panels, show that upon overexpression of SecA, the mass of 51% of the cells (n = 555) increased significantly and they occupied a lemon shape with their central part inflated, whereas the MreB-msfGFP^SW^ appeared largely diffused throughout the cytoplasm. In contrast, when we overexpressed a control protein, LacZ, neither the localization of MreB-msfGFP^SW^ nor the shape of the cells were affected (Fig 2F, lower panels). These results suggest that indeed excess SecA is titrating MreB away from the membrane. However, an alternative explanation could be that high levels of SecA change the stoichiometry of the Sec system, thus exerting a dominant negative effect on the secretion machinery, which might indirectly affect MreB localization. However, the fact that MreB localization appeared diffuse, rather than membrane localized upon SecA overexpression, suggests that this effect is largely direct.

The results presented thus far indicate that the Sec system is important for proper localization of the MreB cytoskeleton. Is the reverse also true, that is, is MreB important for SecA localization? To address this question, we observed the subcellular localization of SecA fused to YFP and expressed from its native chromosomal locus in cells also expressing MreB-RFP^SW^. Cells were either treated with different concentrations of A22 or were not treated by A22, and images were acquired to monitor the localization of MreB and SecA at different time points after treatment. The results show that, in A22-untreated cells, SecA-YFP molecules localized mainly along the membrane (S4 Fig, upper panels). Notably, even in the A22 treated cells, SecA-YFP remained unaffected, whereas MreB-RFP^SW^ localization was clearly affected by A22, in a concentration dependent manner (S4 Fig, center and lower panels). Of note, the effect of A22 on the localization of MreB-msfGFP^SW^ was also similar to that of MreB-RFP^SW^ (S4 Fig, compare right panels to left panels). Our observations are in agreement with a previous report, which suggested that SecA localization in *B*. *subtilis* cells is independent of MreB [40].

Taken together, our results suggest that SecA is a morphogenetic protein that affects MreB localization, as well as cell shape.

### Other components of the Sec system also affect MreB localization

**Fig 3:**
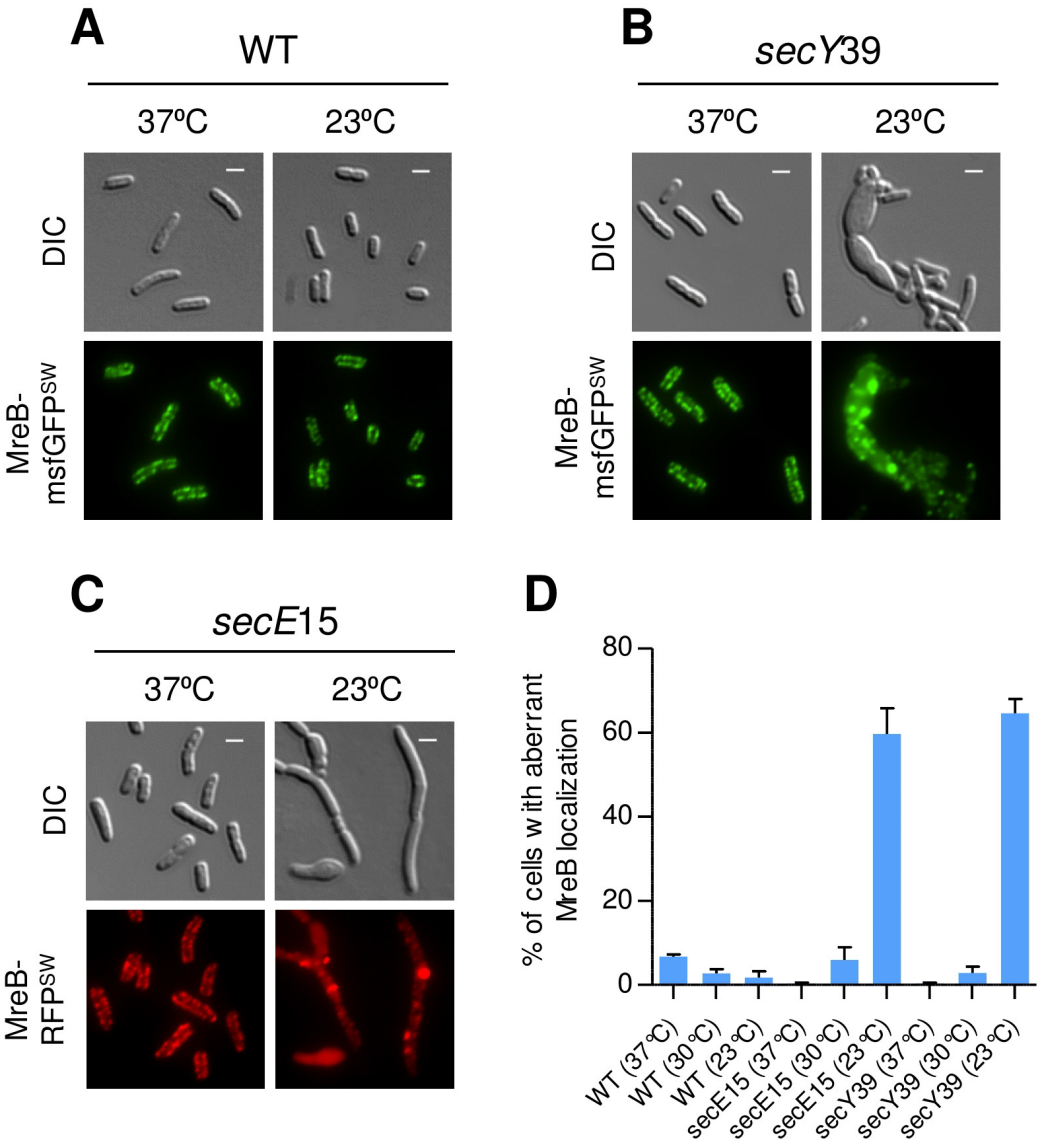
MreB localization is disrupted in SecE and SecY-defective cells. **(A** and **B)** Images of wild-type and *secY*39 (*cs*) mutant cells, which express MreB-msfGFP^SW^ and grown at the permissive (37°C) (left panels) or non-permissive (37°C) temperature (right panels). **(C)** Images of *secE*15 (*cs*) mutant cells, which express MreB-RFP^SW^ and grown at the permissive (37°C) (left panels) or non-permissive (37°C) temperature (right panels). (**D**) Percentage of cells with mislocalized MreB. More than 300 cells from each sample were analyzed from three independent experiments. Means and standard deviations are shown. The GFP and mCherry fusion proteins were observed by fluorescence microscopy and cells were observed with DIC microscopy. Shown are DIC (grey) GFP (green) and mCherry (red) fluorescence signals. Scale bar corresponds to 2 μm.

To check if other components of the Sec system also influence MreB localization, we tested the localization of MreB-msfGFP^SW^ in *secY*39 and in *secE*15 mutant cells. The results show that, while the localization of MreB-msfGFP^SW^ was largely unaffected in wild-type cells grown at the at the permissive (37°C) or restrictive (23°C) temperatures (Fig 3A and 3D), a significant fraction of MreB-msfGFP^SW^ was mislocalized, accumulating in intracellular foci, in *sec*Y39 mutant cells grown at the restrictive temperature (23°C), but not at the permissive temperature (37°C) (Fig 3B and 3D). Similarly, MreB-RFP^SW^ was mislocalized in *secE*15 mutant cells grown at the restrictive temperature (23°C), but not at the permissive temperature (37°C) (Fig 3C and 3D). Consistent with the defect in MreB localization, the shape of SecY- and SecE-impaired cells also appeared abnormal. Of note, unlike *secA*51 cells, which exhibited a defect in MreB localization when grown at the semi-permissive temperature (37°C), the *secY*39 and *secE*15 mutant cells displayed defects in MreB localization only at the restrictive temperature (23°C) and not at the semi-restrictive temperature (30°C) (Fig 3D). This apparently explains why *secY*39 and *secE*15 cells were not sensitive A22 when grown at the semi-restrictive temperature (S1 Fig). The results thus far indicate that components of the Sec system are important for spatial organization of MreB in *E*. *coli* cells.

### SecA is important for cell wall synthesis and impacts cell wall physiological properties

**Fig 4:**
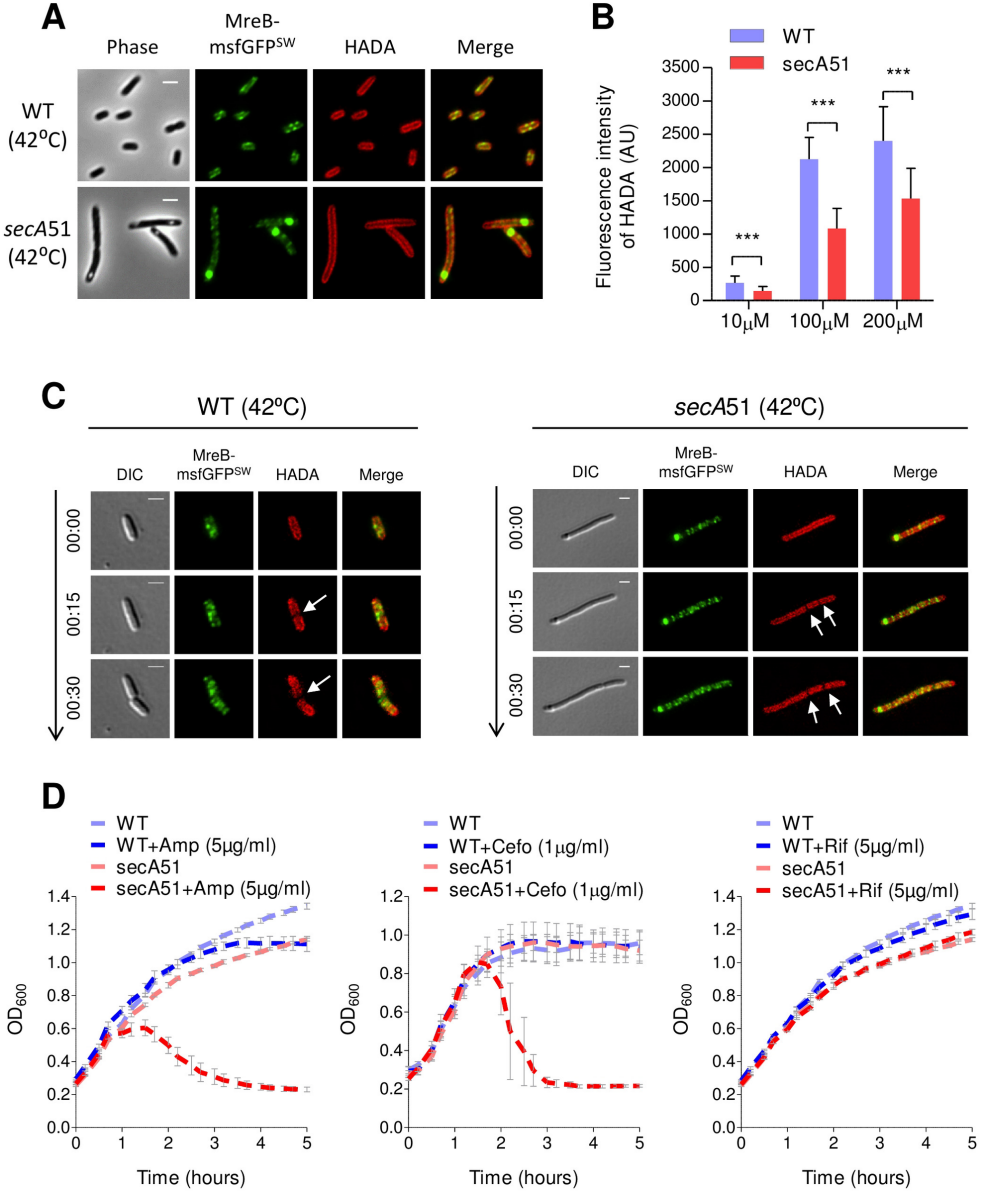
SecA affects cell wall synthesis and its physiological properties. **(A)** Images of wild-type and *secA*51 mutant cells expressing MreB-msfGFP^SW^ and grown at the non-permissive temperature (42°C). Cell wall was visualized by labeling with HADA. **(B)** The bar chart presents the average HADA fluorescence (AU) in wild-type and *secA*51 cells grown at the non-permissive temperature (42°C) and labelled with the indicated concentrations of HADA. More than 60 cells from each sample were analyzed from two independent experiments. Means and standard deviations are shown. The statistical significance was calculated using unpaired t-test analysis (*** p<0.0001). **(C)** Pulse-chase time-lapse microscopy images of HADA-labelled wild-type and *secA*51 mutant cells expressing MreB-msfGFP^SW^ and grown at the non-permissive temperature (42°C) in the absence of the label. Shown are the images obtained at time-points 0, 15 and 30 minutes. Arrows mark the location of new peptidoglycan insertion. **(D)** Sensitivity of wild-type and *secA*51 cultures to sub-inhibitory concentrations of ampicillin (5 μg/ml), cefotaxime (1 μg/ml) or rifampicin (5 μg/ml) at a semi-restrictive temperature (37°C). Values represent the average of three independent experiments. The GFP fusion protein and the HADA label (red) were observed by fluorescence microscopy and cells were observed with DIC or phase microscopy. Merge of the fluorescence signals (GFP, green; HADA, red) is shown for A and C. Scale bar corresponds to 2μm.

Since spatial organization of cell wall synthesis is regulated by MreB, we tested whether inactivation of SecA, shown above to affect MreB localization, also affects cell wall synthesis. For this purpose, we stained the wild-type and *secA*51 cells, which were grown under the restrictive temperature, using fluorescent HCC-amino-D-alanine (HADA) [41]. The results, presented in Fig 4A, show that the localization pattern of the HADA label appeared roughly similar in *secA*51 mutant cells and in wild-type cells. This was expected, since SecA-defective cells contain cell wall, as evidenced by their rod shape rigidity. Still, when we incubated wild-type and SecA-defective cells, grown at the restrictive temperature, with various concentrations of HADA and quantified their average fluorescence intensities after 30 minutes, we found that, the average fluorescence of HADA was significantly lower in SecA-defective cells compared to wild-type cells in all HADA concentrations tested (Fig 4B). This data suggests that inactivation of SecA results in defective cell wall synthesis in these cells.

A recent study has shown that artificial localization of *E*. *coli* MreB to polar regions results in polar cell wall synthesis and formation of ectopic poles [42]. Although ectopic poles are not observed in *secA*51 cells, we asked whether the mislocalized MreB in these cells, which is mainly localized at polar or sub-polar regions, performs polar cell wall synthesis. For this purpose, we stained the wild-type and *secA*51 mutant cells, grown at the restrictive temperature with HADA and performed pulse-chase time-lapse microscopy to visualize the sites of new cell wall synthesis. Notably, the sites at which new cell wall synthesis occur can be identified as regions at which the HADA label disappears. The results show that, in wild-type cell, the HADA label disappeared mainly from mid-cell during 30 minutes of chase at the restrictive temperature and was gradually concentrating in the poles (Fig 4C, left panel). On the other hand, in *secA*51 cells, the HADA label disappeared from random sites along the cell axis and was not accumulating in the poles during the 30 minutes chase (Fig 4C, right panel). This indicates that the mislocalized MreB in SecA-defective cells does not mediate polar cell wall synthesis.

In light of the defects in cell wall synthesis in *secA*51 cells, we speculated that these mutant cells would exhibit altered sensitivity to cell wall-targeting antibiotics compared to wild-type cells. To test this hypothesis, we performed antibiotic-induced cell lysis assay using ampicillin and cefotaxime, which are cell wall-targeting antibiotics. As a control antibiotic that does not target the cell wall, we treated cells with rifampicin, which is a transcription inhibitor. All antibiotics were added in sub-inhibitory concentrations. When incubated with ampicillin or cefotaxime at a semi-restrictive temperature (37°C), the OD_600_ of a *secA*51 culture dropped rapidly indicating cell lysis (Fig 4D, left and middle panels). In contrast, untreated *secA*51 cells, antibiotic-treated or untreated wild-type cells and rifampicin treated cells continued to grow during the course of the experiment (Fig 4D, right panel), suggesting that the *secA*51 cells are hypersensitive to cell wall targeting antibiotics. To directly visualize the effect of ampicillin on the *secA*51 cells, we imaged wild-type and *secA*51 cells grown in the absence or in the presence of a sub-inhibitory concentration of ampicillin at the semi-restrictive temperature. Consistent with the growth inhibition of the *secA*51 cells observed in Fig 4D, lysis of *secA*51 cells, but not of the wild-type cells, was observed within 2 hours after the antibiotic additions (S5 Fig). Together, these results indicate that the synthesis and the physiological properties of the cell wall are severely affected upon disruption of the Sec system.

### Mislocalization of MreB in SecA-deficient cells induces formation of aberrant membrane regions

**Fig 5:**
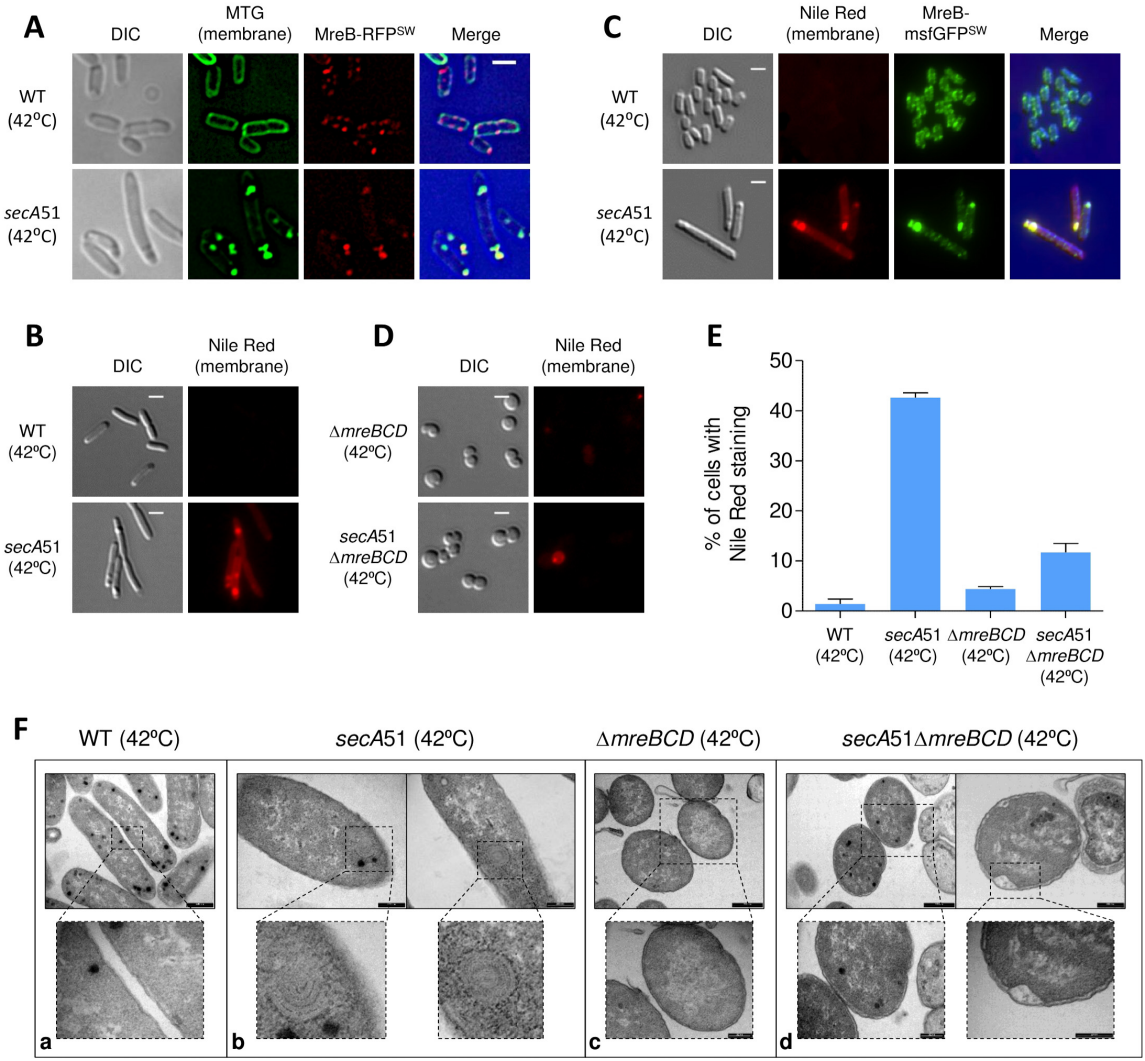
MreB mislocalization causes aberrant membrane organization in *secA* mutant. (**A**) Images of wild-type and *secA*51 mutant cells expressing MreB-RFP^SW^ and grown at the non-permissive temperature (42°C). Membrane was visualized with MTG stain. The mCherry fusion protein and membrane stain were observed by fluorescence microscopy and cells were observed with DIC microscopy. Shown are DIC (grey), MTG (green), mCherry (red), and overlays of the fluorescence signals (MTG and mCherry) over the DIC images (blue background). (**B**) Images of wild-type and *secA*51 mutant cells grown at the non-permissive temperature (42°C). Membrane was visualized with Nile Red stain and observed by fluorescence microscopy (red) and cells were observed with DIC microscopy (grey). (**C**) Images of wild-type and *secA*51 mutant cells expressing MreB-msfGFP^SW^ from the chromosome and grown at the non-permissive temperature (42°C). Membrane was visualized with Nile Red. The GFP fusion protein (green) and the membrane stain (red) were observed by fluorescence microscopy and cells were observed with DIC microscopy (grey). Overlays of the fluorescence signals (GFP and Nile Red) over the DIC images (blue background) are also shown. (**D**) Images of Δ*mreBCD* and *secA*51Δ*mreBCD* mutant cells grown at the non-permissive temperature (42°C). Membrane was visualized with Nile Red stain and observed by fluorescence microscopy (red) and cells were observed with DIC microscopy (grey). (**E**) Percentage of Nile Red-stained cells (wild-type, *secA*51, Δ*mreBCD* and *secA*51Δ*mreBCD*), grown at the non-permissive temperature (42°C). More than 400 cells from each sample were analyzed from two independent experiments. Means and standard deviations are shown. (**F**) Transmission electron micrograph (TEM) images of wild-type (a), *secA*51 (b), Δ*mreBCD* (c) and *secA*51Δ*mreBCD* (d) grown at the non-permissive temperature (42°C). Dotted box indicate the location of magnified images (lower panels). For fluorescence microscopy images, scale bar corresponds to 2 μm. For TEM images, scale bar corresponds to 500 nm or 200 nm.

MreB was suggested to influence membrane organization and membrane protein dynamics [16]. To examine whether the mislocalized MreB in *secA*51 cells affects their membrane organization, we first stained the membrane of wild-type and *secA*51 cells, both expressing MreB-RFP^SW^ and grown at the restrictive temperature, with Mito-tracker green (MTG), which is a lipid-permeable dye. The results in Fig 5A show that, unlike the even staining of the membrane of wild-type cells by MTG, which exhibited the expected punctate pattern of MreB-RFP^SW^ along the cell periphery (Fig 5A, upper panels), the MTG in *secA*51 cells concentrated at polar and sub-polar regions, which are also the sites of MreB-RFP^SW^ accumulation (Fig 5A, lower panels).

Next, we asked if the sites at which the MTG stain concentrated in *secA*51 cells are regions of increased fluidity (RIFs), which are specialized lipid domains formed at the sites of MreB assembly [16]. To check this, we stained the membrane of wild-type and *secA*51 cells, grown at the restrictive temperature, with the membrane fluidity-sensitive dye Nile Red [43]. The results in Fig 5B show that, although the Nile Red was hardly detected at the membrane of wild-type cells under the staining conditions used, it formed bright foci in *secA*51 cells. Of note, unlike *B*. *subtilis*, which can be stained by Nile Red under the conditions used here, wild-type *E*. *coli* cells cannot be stained by the dye (S6 Fig), although the *secA*51 cells were stained (Fig 5B). Moreover, the Nile Red foci co-localized with MreB-msfGFP^SW^, expressed from the native locus, in *secA*51 cells grown at the restrictive temperature (Fig 5C), suggesting that the brightly stained RIFs observed in the *secA*51 cells were formed due to MreB accumulation. To test whether MreB is the cause for the formation of the RIFs in *secA*51 cells, we stained *secA*51*ΔmreBCD* double mutant and *ΔmreBCD* mutant cells grown under *sec*-restrictive conditions with Nile Red and calculated the percentage of cells exhibiting Nile Red staining. The results in Fig 5D and 5E show that the percentage of *secA*51*ΔmreBCD* double mutant cells stained by Nile Red was 3 times lower compared to cells carrying only the *secA*51 mutation (Fig 5E). These results suggest that the RIFs in *secA*51 cells are largely formed in an MreB-dependent manner. Because Nile Red is known to be a substrate for the pmf-driven multidrug efflux in *E*. *coli*, an alternative explanation for the poor staining of wild-type cells could be that they are well energized [44]. However, SecA-defective cells are unlikely to be compromised for their efflux function, since increased staining with Nile Red is reversed when SecA is inactivated in the absence of MreB. To take a closer look at the membrane distortion in *secA*51 cells, we performed TEM analysis on wild-type and *secA*51 mutant cells that were grown at the restrictive temperature. Representative images, shown in the Fig 5Fb, reveal extensive multilayer membrane regions near the poles or at other regions of *secA*51 cells, which were not observed in wild-type cells (Fig 5Fa), suggesting that the intensely stained membrane foci observed in *secA*51 cells by light microscopy at the sites of MreB accumulation are membrane involutions composed of many layers of membrane. Again, these multilayers of membranes are formed in an MreB-dependent manner, since they were not detected in *ΔmreBCD* mutant or *secA*51*ΔmreBCD* double mutant cells grown under *sec*-restrictive conditions (Fig 5Fc and 5Fd).

Together, these results suggest that MreB induces the formation of RIF-rich multilayer membrane regions in cells defective for SecA.

### SecA influences cell division by affecting MreB-FtsZ interaction and Z-ring formation

**Fig 6:**
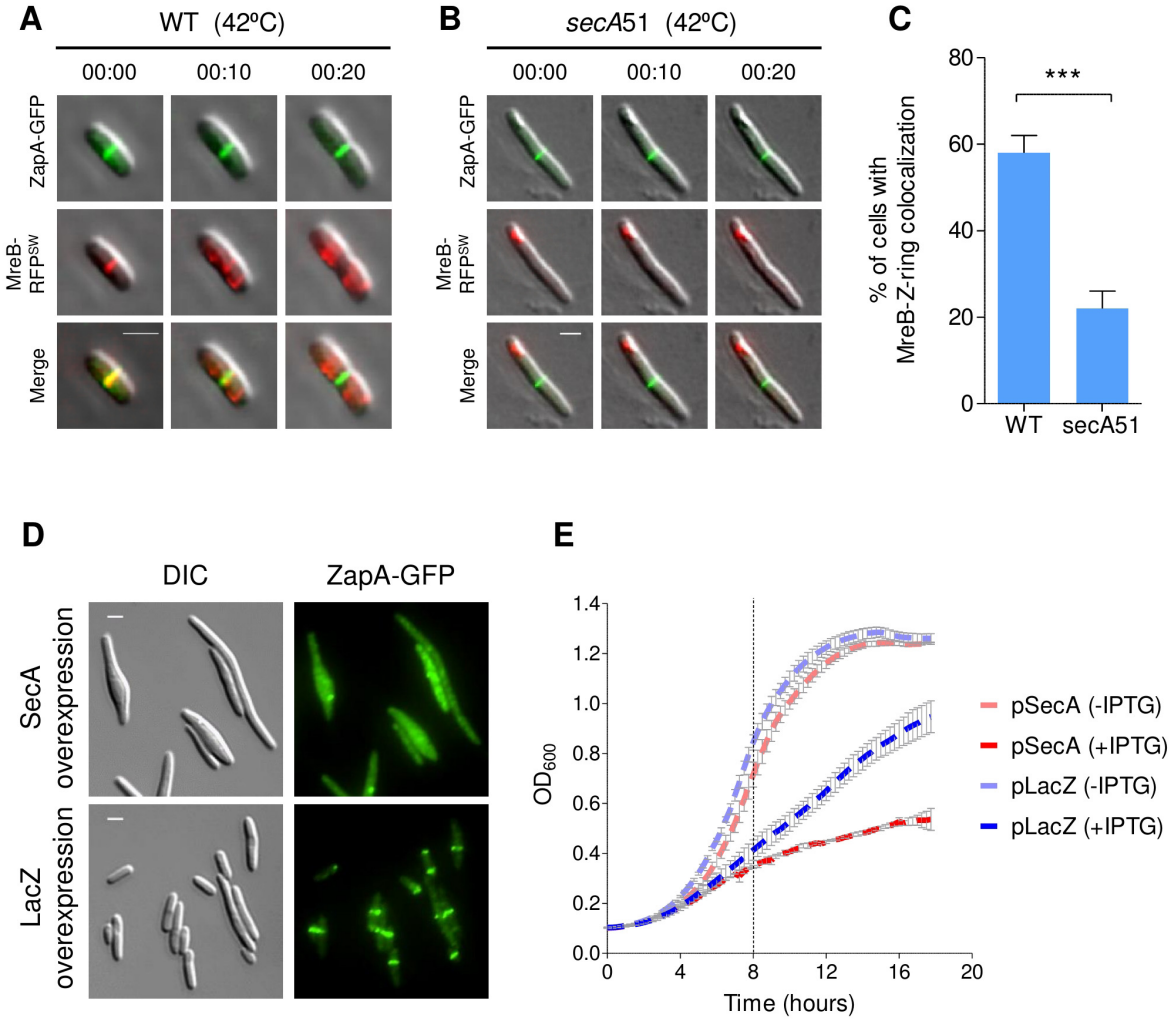
Cell division is affected by SecA disruption. (**A** and **B**) Time-lapse microscopy images of wild-type (A) and *secA*51 cells (B) expressing MreB-RFP^SW^ and ZapA-GFP and grown at the non-permissive temperature (42°C). The mCherry and GFP fusion proteins were observed by fluorescence microscopy and cells were observed with phase microscopy. Shown are the overlays of the fluorescence signals (GFP, green; mCherry, red) over the phase contrast images (grey) obtained at time-points 0 and 13 minutes. Arrows mark co-localization of MreB-RFP^SW^ and ZapA-GFP. (**C**) Percentage of wild-type and *secA*51 cells exhibiting MreB-Z-ring colocalization during growth at the non-permissive temperature (42°C). More than 100 cells from each sample were analyzed from two independent experiments. Means and standard deviations are shown. The statistical significance was calculated using unpaired t-test analysis (*** p<0.0001). (**D**) Images of cells expressing ZapA-GFP from the chromosome and overexpressing wild-type SecA or LacZ from a plasmid. The GFP fusion protein was observed by fluorescence microscopy (green) and cells were observed with DIC microscopy (grey). (**E**) Growth curve of wild-type (MG1655) cells overexpressing (due to the addition of 0.1 mM IPTG) or not overexpressing (no IPTG added) SecA or LacZ. Values represent the average of three independent experiments. Dotted line in the graph indicates the time at which cells were imaged for (D). Scale bar for fluorescence microscopy images correspond to 2 μm.

The *secA*51 cells are filamentous at the restrictive temperature, indicating that their cell division is inhibited. In light of the finding that recruitment of MreB to the septum and its interaction with FtsZ are essential for cell division, together with the demonstration that cells expressing mutant MreB that cannot interact with FtsZ are defective in cell division and form filaments [11], we asked if the mislocalized MreB in *secA*51 cells also fails to be recruited to the Z-ring, thus causing stalling of cell division and cell filamentation. To answer this question, we followed the localization of MreB-RFP^SW^ together with ZapA-GFP in both wild-type and *secA*51 cells grown at the restrictive temperature by time-lapse microscopy. In agreement with previous findings [11], our results show that MreB was detected as co-localizing with the Z-ring in almost 60% of wild-type cells for a brief period of time (less than 3 minutes) prior to being redistributed in the cell periphery (Fig 6A and 6C). In contrast, the sub-polarly accumulated MreB-RFP^SW^ in *secA*51 cells remained mostly static and was detected as co-localizing with the Z-ring in only 25% of the cells (Fig 6B and 6C). Based on these results, we suggest that the inefficient recruitment of MreB to the Z-ring, which affects the interaction of MreB and FtsZ, contributes to the defect in cell division that the *secA*51 cells show.

Having observed that reducing the amount of active SecA precludes MreB-Z-ring association and in light of the known interaction of SecA with FtsZ, we asked if increasing the amount of SecA would also affect Z-ring formation and/or distribution. The results presented in Fig 6D show that, upon overexpression of SecA, ZapA-GFP appears completely diffused in the cytoplasm (Fig 6D, upper panels), whereas overexpression of a control protein, LacZ, did not affect Z-ring distribution (Fig 6D, lower panels). In light of the effects of SecA overexpression on ZapA and MreB localization, we tested if SecA overexpression affects growth rate. The results in Fig 6E show that the growth rate of cells overexpressing SecA was dramatically reduced compared to cells overexpressing LacZ or not overexpressing any of these proteins (Fig 6E).

All in all, the above results suggest that inactivation of SecA affects cell division by perturbing MreB-Z-ring association, while overproduction of SecA affects cell division by leading to Z-ring dispersal.

### Improper targeting of MreB to the membrane in SecA-deficient cells leads to aberrant organization of MreB and defects in cell division

**Fig 7:**
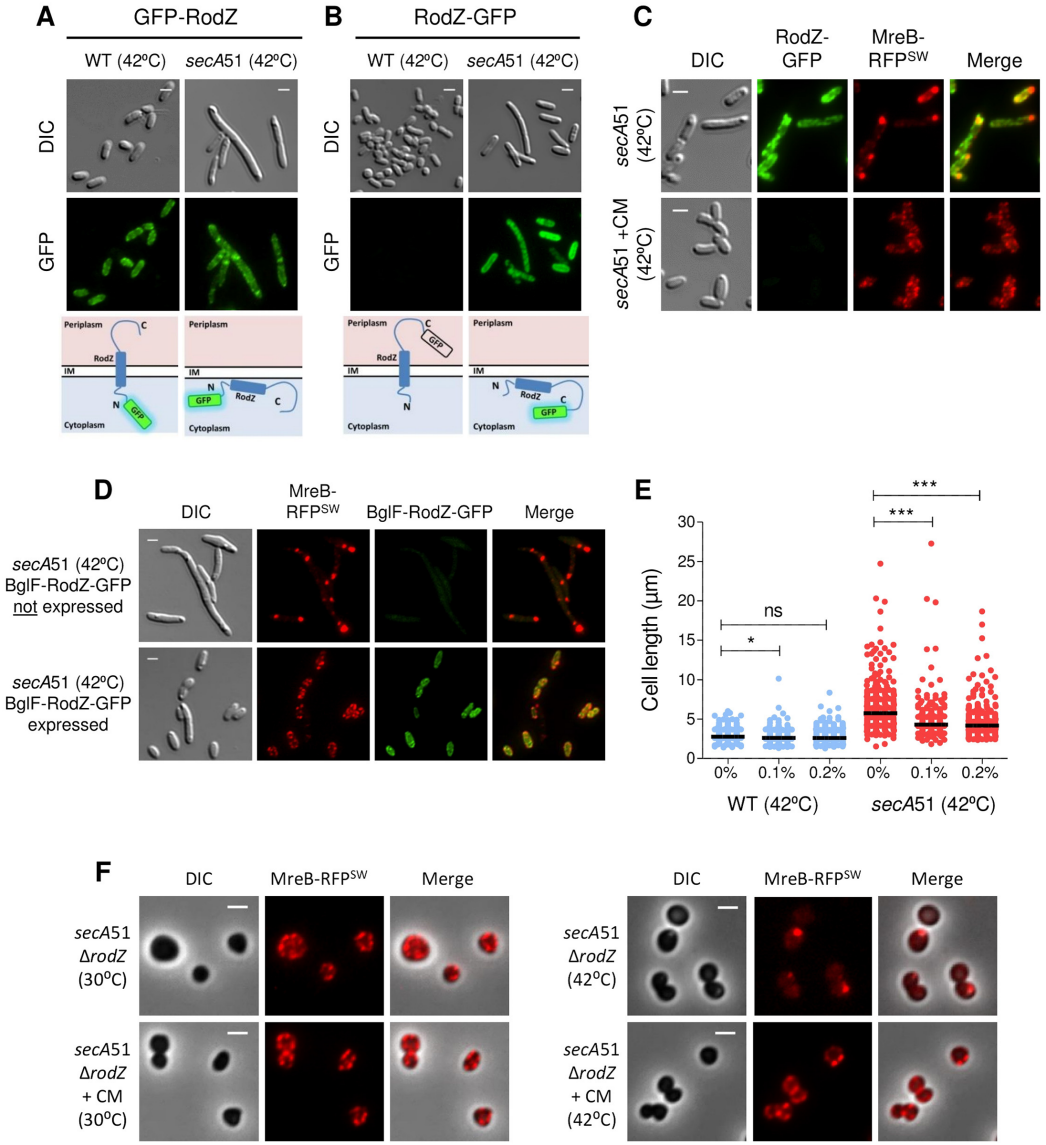
Improper targeting of MreB in SecA-deficient cells results in aberrant organization of MreB and in a division defect. (**A** and **B**) Images of wild-type and *secA*51 mutant cells expressing GFP-RodZ (A) or RodZ-GFP (B) and grown at the non-permissive temperature (42°C). Schematic presentation of GFP-RodZ and RodZ-GFP cellular localization and fluorescence in wild-type and in *secA*51 mutant cells are shown below (A) and (B), respectively. (**C**) Images of *secA*51 mutant cells expressing RodZ-GFP and MreB-RFP^SW^ and grown at the non-permissive temperature (42°C) without (upper panels) or with (lower panels) sub-inhibitory concentration of chloramphenicol (1 μg/ml). (**D**) Images of *secA*51 mutant cells expressing MreB-RFP^SW^, which express or do not express BglF-RodZ-GFP (lower and upper panels, respectively), and grown at the non-permissive temperature (42°C). (**E**) Cell length distribution of wild-type and *secA*51 cells grown at the non-permissive temperature (42°C) and expressing BglF-RodZ-GFP in different arabinose concentrations. The black bar in the data represents the median value of more than 300 from each sample obtained from two independent experiments. The statistical significance was calculated using unpaired t-test analysis (* p<0.05; *** p<0.0001). (**F**) Images of *secA*51 Δ*rodZ* mutant cells expressing MreB-RFP^SW^ and grown at the permissive (30°C) or non-permissive temperature (42°C) without (upper panels) or with (lower panels) sub-inhibitory concentration of chloramphenicol (1 μg/ml). The mCherry and GFP fusion proteins were observed by fluorescence microscopy and cells were observed with DIC or phase microscopy. Shown are DIC (grey), mCherry (red) and GFP (green) fluorescence signals. Merge of the GFP and mCherry signals are shown for (C) and (D). Overlays of the fluorescence signals (mCherry) over the phase images (grey background) are shown for (F). Scale bar corresponds to 2 μm.

Our findings, which imply that the Sec system is important for the localization and functioning of MreB, raise the question of what is the molecular basis of MreB mislocalization in *secA*51 cells. RodZ, which is important for localizing MreB near the membrane and linking it to the cell wall synthesizing proteins [35,45,46], was recently shown to be inserted into the membrane by the Sec system [47] and could account for MreB mislocalization in cells with impaired Sec system. To validate that RodZ is not targeted to the membrane in Sec-deficient cells, we took advantage of RodZ membrane topology in the following way. Since the N-terminus (N-ter) of RodZ is in the cytoplasm, whereas its C-terminus (C-ter) is in the periplasm, only GFP fused to its N-ter is expected to be fluorescent, because regular GFP does not fluoresce in the periplasm [38,48]. We therefore fused GFP to the N-ter or C-ter of RodZ and, after confirming the ability of both fusions to complement RodZ-deficient cells, monitored their fluorescence/localization in wild-type and *secA*51 cells grown at the restrictive temperature. GFP fused to RodZ N-ter (GFP-RodZ) formed a typical spotty helix-like distribution pattern when expressed in wild-type cells (Fig 7A, left panel), whereas in *secA*51 cells it exhibited a mixture of the typical spotty helix-like distribution, as well as clusters of aberrantly localized protein (Fig 7A, right panel). However, GFP fused to RodZ C-ter (RodZ-GFP) was not fluorescent at all when expressed in wild-type cells, (Fig 7B, left panel), consistent with the presence of GFP in the periplasmic space, not enabling its proper folding. When expressed in *secA*51 cells grown at the restrictive conditions, RodZ-GFP was fluorescent and formed a spotty localization pattern (Fig 7B, right panel), indicating that RodZ is not inserted into the membrane and its C-ter does not reach the periplasm. These results confirm that RodZ requires a functional Sec system for its membrane insertion. Of note, RodZ and MreB exhibit different distribution patterns in SecA-inactivated cells, as indicated by imaging RodZ-GFP together with MreB-RFP^SW^ in *secA*51 cells at the restrictive temperature (Fig 7C, upper panels), suggesting that their association is affected in the absence of functional SecA.

Previous studies have shown that partial suppression of translation, which reduces the level of proteins that need to be secreted, can rescue the secretion defect in *secA*51 cells [49]. To confirm that the defect in protein secretion is the reason for RodZ and MreB mislocalization in *secA*51 cells, we asked if the localization pattern of RodZ-GFP and MreB-RFP^SW^ can be restored in *secA*51 cells grown at the restrictive temperature, by treating them with a sub-inhibitory concentration of the translation inhibitor chloramphenicol. The results in Fig 7C, lower panels, demonstrate that wild-type-like patterns were indeed restored for both MreB-RFP^SW^ and RodZ-GFP in *secA*51 cells grown at the restrictive temperature after treating them with CM, Proper localization of RodZ in these cells is evident from the lack of GFP signal. Taken together, both MreB and RodZ fail to localize in *secA*51 cells, but when the burden on the Sec system is partially relieved, localization of both proteins is restored.

Next, we asked if the defect in MreB localization in SecA-deficient cells can be restored by rerouting RodZ to the membrane in a SecA-independent manner. For this purpose, we constructed a RodZ hybrid protein, which is fused at its C-ter to GFP and at its N-ter to the β-glucoside permease BglF, an integral membrane protein that lacks a signal sequence, as predicted by SPOCTOPUS [50] and SignalP 4.1 [51] (which predict the Sec-type signal-anchor within a protein sequences). Importantly, BglF-like permeases of the phosphotransferase system (PTS) were shown to be inserted into the membrane independently of SecA [52]. We then tested if this chimeric protein can recruit MreB to the membrane in SecA-impaired cells. The results in Fig 7D demonstrate that the C-ter of RodZ, which contains its membrane-insertion domain (a single α helix) and is fused to GFP, although brought by BglF to the cell periphery, does not reach the periplasm, but, rather, remains in the cytoplasm, as indicated by the fluorescence of the BglF-RodZ-GFP fusion expressed from the tight Ara promoter (Fig 7D, bottom panels). Nevertheless, upon overexpression of the BglF-RodZ-GFP fusion by the addition of 0.1% arabinose or more, the localization pattern of the chromosomally expressed MreB-RFP^SW^ in *secA*51 cells grown at 42°C was restored, as opposed to its mislocalization when expression of the BglF-RodZ-GFP fusion in these cells was not induced (Fig 7D, upper panels). These results imply that the overproduced RodZ, which was brought near the membrane by BglF, although not properly inserted into it, restored the wild-type-like localization of MreB in the absence of SecA. Notably, MreB has been shown to interact with the cytoplasmic domain of RodZ [35,53], explaining why the improperly membrane-inserted RodZ fusion protein can still interact with MreB.

Intriguingly, not only was localization of MreB restored in these cells, but the filamented phenotype of the *secA*51 cells was significantly reduced in a BglF-RodZ-GFP concentration dependent manner (Fig 7E). While overexpression of BglF-RodZ-GFP ameliorated the cell division defect of *secA*51 cells, without significantly affecting the wild-type cells (Fig 7E), it did not restore their viability at the non-permissive temperature (S7 Fig), indicating that partial restoration of MreB localization did not solve the secretion defect.

To determine whether RodZ alone contributes to MreB mislocalization in SecA-defective cells, we constructed a Δ*rodZ secA*51 strain, which expresses MreB-RFP^SW^, and monitored the localization of MreB under *sec*-restrictive and permissive conditions. Interestingly, inactivation of SecA in Δ*rodZ* cells still affected MreB localization, as evidenced by the presence of aberrant MreB clusters in these cells (Fig 7F). These aberrant MreB clusters are not formed due to the absence of RodZ, since MreB localization appeared similarly in Δ*rodZ secA*51 cells, grown under permissive condition, with and without sub-inhibitory concentration of chloramphenicol, or grown under Sec depletion condition, with sub-inhibitory concentration of chloramphenicol (Fig 7F). This implies that SecA affects MreB localization via RodZ-dependent and RodZ-independent pathways.

### The dependence of MreB localization on SecA is conserved in *Caulobacter crescentus*

**Fig 8:**
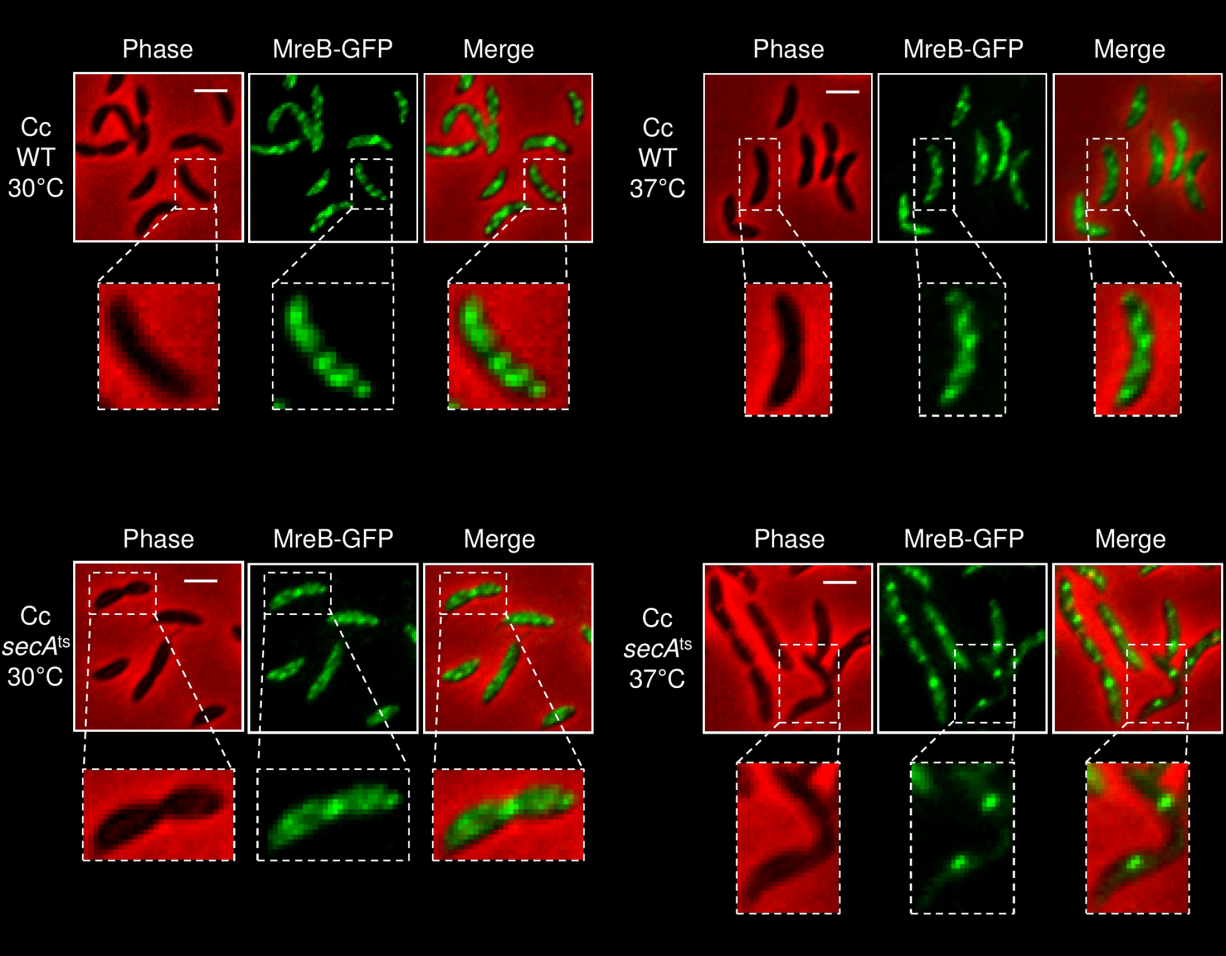
SecA inactivation affects MreB localization in *C*. *cresentus*. Images of wild-type and *sec*A^ts^ mutant cells expressing GFP-MreB and grown at the permissive (30°C) (left panels) or restrictive (37°C) temperature (right panels). The GFP fusion protein was observed by fluorescence microscopy and cells were observed with phase microscopy. Shown are phase (red), GFP (green) and overlays of the fluorescence signals (GFP) over the phase images (red background). Dotted boxes indicate the location of magnified images shown below. Scale bar corresponds to 2 μm.

Both the Sec system and the MreB cytoskeletal system are highly conserved across bacterial species, with MreB being largely conserved in rod-shaped bacterial cells. To test whether SecA-controlled localization of MreB is a conserved mechanism in bacteria, we examined the localization of MreB in *Caulobacter cresentus* cells defective in functional SecA. Of note, *C*. *crescentus* also has a RodZ protein which is important for the localization of MreB [46]. To visualize MreB, we integrated a plasmid that expresses MreB-GFP under the control of xylose promoter into the chromosome of LS107, a wild-type *C*. *crescentus* strain, and of LS416, a *secA*^ts^ mutant *C*. *crescentus* strain, which is defective in SecA activity when grown at non-permissive temperature (37°C). When wild-type or *secA*^ts^ mutant cells were grown under the permissive temperature (30°C), localization of MreB-GFP appeared normal in both cell types, as indicated by the formation of spotty or medial localization pattern (Fig 8, left panels). However, when grown at the non-permissive temperature (37°C), localization of GFP-MreB, which remains unaffected in wild-type cells, was severely affected in *secA*^ts^ mutant cells (Fig 8, right panels). Thus, rather than forming a wild-type-like localization patterns, MreB-GFP formed multiple distinct puncta along the *secA*^ts^ mutant cells. Of note, in accordance with a previous report [54], the *secA*^ts^ mutant cells that were grown at the non-permissive temperature were elongated, due to inhibition of cell division, indicating that SecA is inactivated in these cells. Hence, our data suggest that SecA-dependent localization of MreB is conserved in gram negative bacteria that diverged one billion years ago.

## Discussion

Central systems are known to cooperate in cell organization, *e*.*g*., in rod-shaped bacteria, interaction of the actin homolog MreB and the tubulin homolog FtsZ is important for cell division [11]. In this study, we uncovered a previously uncharacterized genetic linkage between the genes encoding SecA and MreB, as well as cooperation between the Sec and the MreB cytoskeletal system in bacterial cell organization. Our data indicate that the Sec system is important for proper filament organization and function of MreB. Thus, in *secA*51 cells, which produce non-functional SecA protein at the restrictive temperature, an average of 25% of the total cellular MreB molecules mislocalize as large clusters near the cell poles. The mislocalized MreB clusters either remain static or are repositioned to sub-polar regions. Mislocalization of MreB was also observed in *C*. *crescentus* cells defective in SecA, indicating that the linkage between the two systems is conserved across bacterial species. Still, MreB mislocalization in *C*. *crescentus* cells does not appear similar to that of *E*. *coli* cells possibly due to the different patterns of MreB localization in these two widely diverged gram negative bacterial species [1,13,36]. We further show that mislocalization of MreB in Sec-defective *E*. *coli* cells largely compromises its activity in cell wall synthesis, cell division and membrane organization. Finally, we present evidence that mislocalization of MreB in Sec-defective cells is due to improper positioning of MreB around the cell circumference.

One of the important functions of MreB is spatial coordination of cell wall synthesis. It does so by transiently interacting with and relocating the PG synthesizing proteins around the cell circumference, which result in cell elongation (reviewed in [55,56]); at the same time the physical force generated by the insertion of new cell wall segments drives MreB movement [5,6,7]. Our results show that cell wall synthesis is affected in SecA-inactivated cells, as implied by the reduced incorporation of the fluorescent D-amino acid HADA and by the formation of envelope bulges at the regions where MreB accumulates, which were shown to form as a result of disorganized PG synthesis [57,58]. The hypersensitivity of Sec-defective cells to cell wall targeting antibiotics suggest that in addition to impairment of the synthesis of cell wall, its physiological properties are also affected upon SecA-inactivation. One question that arises is why the *secA*51 cells, grown at the restrictive temperature, which has a moderate growth defect, possibly due to reduced cell wall synthesis, do not adopt a round morphology. The explanation might be provided by the fraction of cellular MreB that retain wild-type-like localization patterns and dynamics, which we observed in SecA-inactivated cells. This fraction might be composed of MreB molecules that were synthesized and localized prior to the inactivation of the Sec machinery. In any case, this fraction may drive basal cell wall synthesis, which, in turn, would account for rod shape maintenance in *secA* mutant cells.

For nearly three decades, inactivation of the Sec system was known to affect membrane biogenesis and induce the formation of intra-cytoplasmic multilayer membranes [59,60]. However, the molecular mechanism/s that underlie this process remained unclear. Our results point at mislocalization of the MreB cytoskeleton as the cause for the formation of such multilayer membranes regions in Sec-impaired cells, since they were not formed in *secA*51Δ*mreBCD* cells grown at *sec*-restrictive conditions. Intriguingly, depletion or overexpression of MreB were previously shown to correlate with the formation of intra-cytoplasmic membrane-bound compartments apparently due to loss of correlation between membrane and cell wall synthesis, which leads to the generation of excess membrane that folds inward [14,33,61]. Hence, our finding that mislocalization of MreB due to Sec inactivation, which results in accumulation of multilayer membrane regions, might very well be also due to the disruption of the balance between the rate of membrane and cell wall synthesis. Of note, MreB-dependent formation of multilayer membrane regions in Sec-defective cells does not contradict the occurrence of other membrane defects in these cells that do not depend on MreB. Our observations are in agreement with a recent study, which suggested that the MreB is a membrane organizer, since aberrant localization of MreB distorts the membrane [16]. The same study also documented association between MreB and RIFs, which are specialized membrane domains with increased fluidity, consistent with our observation that RIFs are formed at regions of MreB accumulation in Sec-defective cells.

Our results on MreB mislocalization in *sec*-defective cells also shed light on the cell division defect of these cells, which leads to their filamentation. In general, inhibition of the Sec system can affect cell division by blocking membrane translocation of Sec-dependent divisome proteins. Potential candidates include FtsQ and EnvC, which were shown to be inserted into the membrane and transported to the periplasm by the Sec system, respectively [62,63] and FtsK, which contains a potential signal sequence (as predicted by SPOCTOPUS [50]). However, our results point at MreB, which has been shown to be recruited to the forming septum in *E*. *coli* by FtsZ during the initial stages of cell division to enable Z-ring constriction, divisome maturation and septal PG synthesis [11], as an additional and early contributor of cell division defect in these cells. Noticeably, we show that by bypassing the dependence of MreB on the Sec system for membrane localization, the cell division defect of SecA-defective cells is partially corrected, suggesting that SecA-MreB cooperation is important for cell division.

Our observation that overexpression of SecA affects MreB subcellular distribution, as well as Z-ring formation, suggests that SecA is a morphogenetic modulatory protein that interacts with central morphogenetic components of the cell, in this case the MreB cytoskeleton or components of the divisome, and affect their organization and thereby cell shape. In line with its unexplored morphogenetic function, a recent study has identified SecA as an important factor necessary for membrane targeting of DivIVA, a *B*. *subtilis* polarity-establishing protein [27].

Our data indicate that SecA-mediated targeting of MreB occurs via RodZ-dependent as well as RodZ-independent pathways. Currently, the mechanism involved in RodZ-independent targeting of MreB is not known. Yet, this pathway appears to be Sec-dependent, since partial suppression of translation, which relieves the secretion defect in SecA-inactivated cells, rescues MreB localization in the absence of RodZ. Having said that, the possibility of direct involvement of SecA in MreB localization cannot be completely ruled out.

Finally, the fact that the cellular concentration of SecA is non-proportionally high, compared to that SecYEG (approximately 13,000 compared to 500 copies per cell, respectively) [22], together with the observation that proteins without a typical signal sequence, such as MreB, FtsZ and DivIVA, interact with SecA and depend on it for their proper localization, supports the idea that SecA has novel, previously unknown functions in cell organization.

## Materials and methods

### Bacterial strains and growth media

Strains and plasmids used in this study are listed in supplementary S1 Table. Overnight *E*. *coli* cultures were grown in LB or M9 glycerol, depending on the experiment, supplemented with appropriate antibiotics. *C*. *crescentus* cultures were grown in PYE medium. Unless the strain was cold sensitive, overnight cultures were grown at 30°C. When appropriate, antibiotics for *E*. *coli* cultures were added at the following concentrations: ampicillin (100 μg/ml), kanamycin (30 μg/ml), chloramphenicol (25 μg/ml) or tetracycline (20 μg/ml) (Sigma-Aldrich). For *C*. *crescentus*, kanamycin was added at a concentration of 5 μg/ml (for liquid media) or 25 μg/ml (for solid media). Unless indicated, M9 media supplemented with glycerol (0.2%) was used for all microscopy experiments.

### Fluorescence microscopy

Fluorescence microscopy was carried out as described previously [64]. In brief, 0.5 ml cells were centrifuged, washed with 1X phosphate buffered saline (PBS) and finally resuspended in 10–100 μl of PBS. Cell suspensions were placed on 1% M9 glycerol agarose pads with uncoated cover-slips or on poly-lysine coated coverslips. *C*. *crescentus* cells were placed on 1% PYE agarose pads with uncoated cover-slips. The membrane was stained with Mito Tracker Green (MTG; Molecular Probes, Invitrogen) at a final concentration of 10 μM. For Nile Red staining, cells were washed and resuspended in 1X PBS that contained 2 μg/ml of Nile Red and incubated at 37°C for 2 minutes. Cells were washed twice before microscopic examination. For staining the cell wall, fluorescent HCC-amino-D-alanine (HADA) was used as described in Supplementary Experimental Procedures (S1 Text).

Cells were visualized and photographed using an Axiovert 200M (Zeiss) inverted microscope equipped with CoolSnap HQ camera (Photometrics, Roper Scientific) or Nikon Eclipse Ti-E inverted microscope equipped with Perfect Focus System (PFS) and ORCA Flash 4 camera (Hamamatsu photonics). Time-lapse imaging was performed using Nikon Eclipse Ti-E equipped with OKOLAB cage incubator. Unless indicated, cells were spotted in 1% M9 glycerol pads which had been pre-equilibrated to the appropriate temperature and imaged by time-lapse microscopy at the respective temperature. Images were processed using Metamorph (Molecular devices) or NIS Elements-AR software. Statistical analyses were performed using GraphPad Prism.

## Supporting information

S1 Table Bacterial strains used in this study and their relevant phenotype. (DOCX)

S2 Table Plasmids used in this study and the proteins they encode. (DOCX)

S3 Table Primers used in the study. (DOCX)

S1 Fig SecE- and SecY-defective cells are not sensitive to A22. Pictures of wild-type, *secE*15^cs^ and *secY*39^cs^ cells spotted after serial dilutions on LB plates with or without sub-inhibitory concentration of A22 (1 μg/ml) and grown for 20 hours at the permissive (37°C), semi-permissive (30°C) or non-permissive (23°C) temperatures.
(TIF)

S2 Fig Western blot analysis of wild-type and *secA*51 mutant cells expressing MreB-msfGFP^SW^ using α-GFP antibodies. Cells were grown at the permissive (30°C) or restrictive (42°C) temperatures and equal amount of samples were collected at specific time points (0h, 1h, 2h and 3h). Samples were separated on 10% SDS polyacrylamide gel, blotted onto nitrocellulose membrane and detected by α-GFP antibodies.
(TIF)

S3 Fig MreB is mislocalized upon SecA depletion. (**A**) Images of wild-type and *secA*51 mutant cell, which express MreB-RFP^SW^, grown at the permissive (30°C), semi-restrictive (37°C), or restrictive (42°C) temperatures. (**B**) Time-lapse microscopy images of *secA*51 mutant cells, which express MreB-msfGFP^SW^, shifted from the non-permissive temperature (42°C) to the permissive temperature (30°C). (**C**) Images of wild-type and *secA*51 mutant cell, which express MalE-sfGFP, grown at the non-permissive temperature (42°C). **(D)** Images of *secA*51cells, which express MreB-msfGFP^SW^ from the chromosome and wild-type SecA or LacZ from a plasmid, grown at the non-permissive temperature (42°C). (**E**) Images of IPTG-controlled *secA* strain (DRH729) expressing MreB-RFP^SW^ in the presence or absence of IPTG. The mCherry and GFP fusion protein were observed by fluorescence microscopy (red and green, respectively), and cells were observed with DIC microscopy (grey). Scale bar corresponds to 2 μm.
(TIF)

S4 Fig SecA localization is not affected by MreB. Images of cells expressing MreB-RFP^SW^ and SecA-YFP (together) or MreB-msfGFP^SW^ and untreated (upper panels) or treated with 5 μg/ml of A22 (middle panels) or 50 μg/ml of A22 (lower panels). The mCherry, GFP and YFP fusion proteins were observed by fluorescence microscopy (red, green and yellow, respectively) and cells were observed with phase microscopy (grey). Merges of the YFP and mCherry fluorescence signals are also shown.
(TIF)

S5 Fig Ampicillin treatment lyses SecA-defective cells. Images of wild-type and *secA*51 cells grown with or without ampicillin at a semi-restrictive temperature (37°C). Dotted red boxed images indicate cell lysis. Cells were observed with phase microscopy (grey). Scale bar corresponds to 2 μm.
(TIF)

S6 Fig Nile Red staining of wild-type *E*. *coli* and *B*. *subtilis* cells. Images of *E*. *coli* MG1655 (upper panel) and *B*. *subtilis* PY79 cells (lower panel) stained with the fluidity-sensitive dye Nile Red. Staining by Nile Red was observed by fluorescence microscopy (red) and cells were observed with DIC microscopy (grey). Scale bar corresponds to 2 μm.
(TIF)

S7 Fig BglF-RodZ-GFP does not rescue the growth defective phenotype of *secA*51 cells. Pictures of wild-type (MG1655) and *secA*51 cells, containing a plasmid that expresses BglF-RodZ-GFP from the Ara promoter, which were streaked on LB plates and incubated at the non-permissive (42°C) temperature for overnight. The concentration of the arabinose added to each plate are indicated.
(TIF)

S1 Movie Time-lapse movie of wild-type cells producing MreB-msfGFP^SW^ and grown at the non-permissive temperature (42°C). Time-lapse images were acquired every 3 minutes for a total of 3 hours.
(MP4)

S2 Movie Time-lapse movie of *secA*51 cells producing MreB-msfGFP^SW^ and grown at the non-permissive temperature (42°C). Time-lapse images were acquired every 3 minutes for a total of 3 hours.
(MP4)

S3 Movie Time-lapse movie of *secA*51 cells producing MreB-msfGFP^SW^ and grown at the non-permissive temperature (42°C). Time-lapse images were acquired every 10 seconds for a total of 10 minutes.
(MP4)

S4 Movie Time-lapse microscopy movie of *secA*51 mutant cells, which express MreB-msfGFP^SW^, shifted from the non-permissive temperature (42°C) to the permissive temperature (30°C). Time-lapse images were acquired every 3 minutes for a total of 90 minutes.
(MP4)

S1 Text Supplemental Experimental Procedures. (DOCX)
