# Supplementary material for: The bacterial Sec system is required for the organization and function of the MreB cytoskeleton
Source: PLoS Genet. 2017 Sep 25;13(9):e1007017. doi: 10.1371/journal.pgen.1007017 (PMC5629013; doi:10.1371/journal.pgen.1007017)
Supplement: S1 Table — (DOCX) [file pgen.1007017.s001.docx]

**Table S1.** Bacterial strains used in this study and their relevant phenotype

| **Strains** | **Relevant genotype** | **Source / Reference** |
| --- | --- | --- |
| MG1655 | F^-^ *λ^-^ ilvG- rfb-50 rph-1* | Lab collection |
| CWB213 | MC4100, *secA*51(Ts) *leuB*::Tn*10* | [1] |
| NO50 | MG1655, *mreB’-msfGfp-‘mreB* *csrD(::neo)* | [2] |
| SUT202 | NO50, *secA*51(Ts) *leuB*::Tn*10* | This study |
| SUT101 | MG1655, *secA*51(Ts) *leuB*::Tn*10* | This study |
| FB76 | TB28, *mreB’-mCherry-‘mreB* *yhdE(::cat)* | [3] |
| SUT102 | FB76, *secA*51(Ts) *leuB*::Tn*10* | This study |
| SUT103 | FB76 evicted of ‘*cat* cassette’ | This study.  Same as FB83 from [3] |
| SUT104 | SUT102 evicted of ‘*cat* cassette’ | This study |
| JW2500-1 | Δ*yfgA762::kan* | CGSC#: 9991  [4] |
| SUT104::∆rodZ | SUT104, Δ*yfgA762::kan* | This study |
| HC261 | TB28, *zapA-gfp(::cat)* | [5] |
| SUT106 | SUT103, *zapA-gfp(::cat)* | This study |
| SUT107 | SUT104, *zapA-gfp(::cat)* | This study |
| PA340-678 | *gltB^+^* ∆*mre-678* | [6] |
| SUT109 | PA340-678, *secA*51(Ts) *leuB*::Tn*10* | This study |
| SX1061 | *secA791-YFP(::cat)* | CGSC #12616.  [7] |
| SUT110 | SUT103, *secA791-YFP(::cat)* | This study |
| PS266 | *zijRK498::Tn5, secE15(Cs)* | CGSC #7144.  (Jonathan Beckwith) |
| SUT113 | PS266, *mreB’-mCherry-‘mreB* *yhdE(::cat)* | This study |
| RO257 | MC4100, *secY39 (Cs)* zhc::Tn10 | Thomas Silhavy |
| LG809 | MC4100, *prlD*21 | Thomas Silhavy |
| SUT114 | RO257, *mreB’-mCherry-‘mreB* *yhdE(::cat)* | This study |
| DRH729 | MC4100 lambda(att)::(P_lac_-_DSW204_-SecA(WT) AmpR | Damon Huber |
| SUT115 | DRH729, *mreB’-mCherry-‘mreB* *yhdE(::cat)* | This study |
| LS107 | NA1000, ∆*bla* | [8] |
| LS416 | LS107, *secA*1 | [8] |
| LS107::MreB | LS107, *pXyl::gfp-mreB* | This study |
| LS416::MreB | LS416, *pXyl::gfp-mreB* | This study |
| PY79 | Wild-type *B. subtilis* | Lab collection |
