# Supplementary material for: The bacterial Sec system is required for the organization and function of the MreB cytoskeleton
Source: PLoS Genet. 2017 Sep 25;13(9):e1007017. doi: 10.1371/journal.pgen.1007017 (PMC5629013; doi:10.1371/journal.pgen.1007017)
Supplement: S2 Table — (DOCX) [file pgen.1007017.s002.docx]

**Table S2.** Plasmids used in this study and the proteins they encode

| **Plasmids** | **Encoded protein(s) / Inducer** | **Reference** |
| --- | --- | --- |
| pLac::MalE-sfGFP | MalE-sfGFP / IPTG-inducible | [9] |
| pCA24N-secA | SecA / IPTG-inducible | [10] |
| pCA24N-lacZ | LacZ / IPTG-inducible | [10] |
| pGS::N-mGFP | mGFP/ IPTG-inducible | This study |
| pGS::mGFP-RodZ | mGFP-RodZ/ IPTG-inducible | This study |
| pBAD-RodZ-GFP | RodZ-GFP/ Ara-inducible | This study |
| pBAD-BglF-RodZ-GFP | BglF-RodZ-GFP/ Ara-inducible | This study |
| LS3813 | *Pxyl::gfp-mreB/*xylose-inducible (used for MreB localization in *C. crescentus*) | [11] |
