# Supplementary material for: The bacterial Sec system is required for the organization and function of the MreB cytoskeleton
Source: PLoS Genet. 2017 Sep 25;13(9):e1007017. doi: 10.1371/journal.pgen.1007017 (PMC5629013; doi:10.1371/journal.pgen.1007017)
Supplement: S3 Table — (DOCX) [file pgen.1007017.s003.docx]

**Table S3.** Primers used in the study

| **Primers** | **Sequence** |
| --- | --- |
| F-BamHI-mGFP-N | G**GGATCC**GAATGAGTAAAGGAGAAGAACTTTTCACTGG |
| R-SacI-mGFP-N | G**GAGCTC**AGCTGCAGCTTTGTATAGTTCATCCATGCCATGTG |
| F-SacI-rodZ | G**GAGCTC**ATGAATACTGAAGCCACGCAC |
| R-XmaI-rodZ | GA**CCCGGG**TTACTGCGCCGGTGATTGTTC |
| F-NheI-rbs-notI-rodZ | AAGTT **GCTAGC** AAGAAGGAGA GCGGCCGCG ATGAATACTG AAGCCACG |
| R-sacI-rodZ-TAA- | AAAT **GAGCTC** CTGCGCCGGTGATTGTTCGGC |
| F-NheI-rbs-bglF | TAT **GCTAGC** AGCGGTAGAGGGCAAGTTATGACGG |
| R-NotI-bglF-full(TAA-) | TAATA**CGCGGCCGC**GCGAATGATGGATAACAGCGGTTC |
