# Supplementary figures and images for: The bacterial Sec system is required for the organization and function of the MreB cytoskeleton
Source: PLoS Genet. 2017 Sep 25;13(9):e1007017. doi: 10.1371/journal.pgen.1007017 (PMC5629013; doi:10.1371/journal.pgen.1007017)

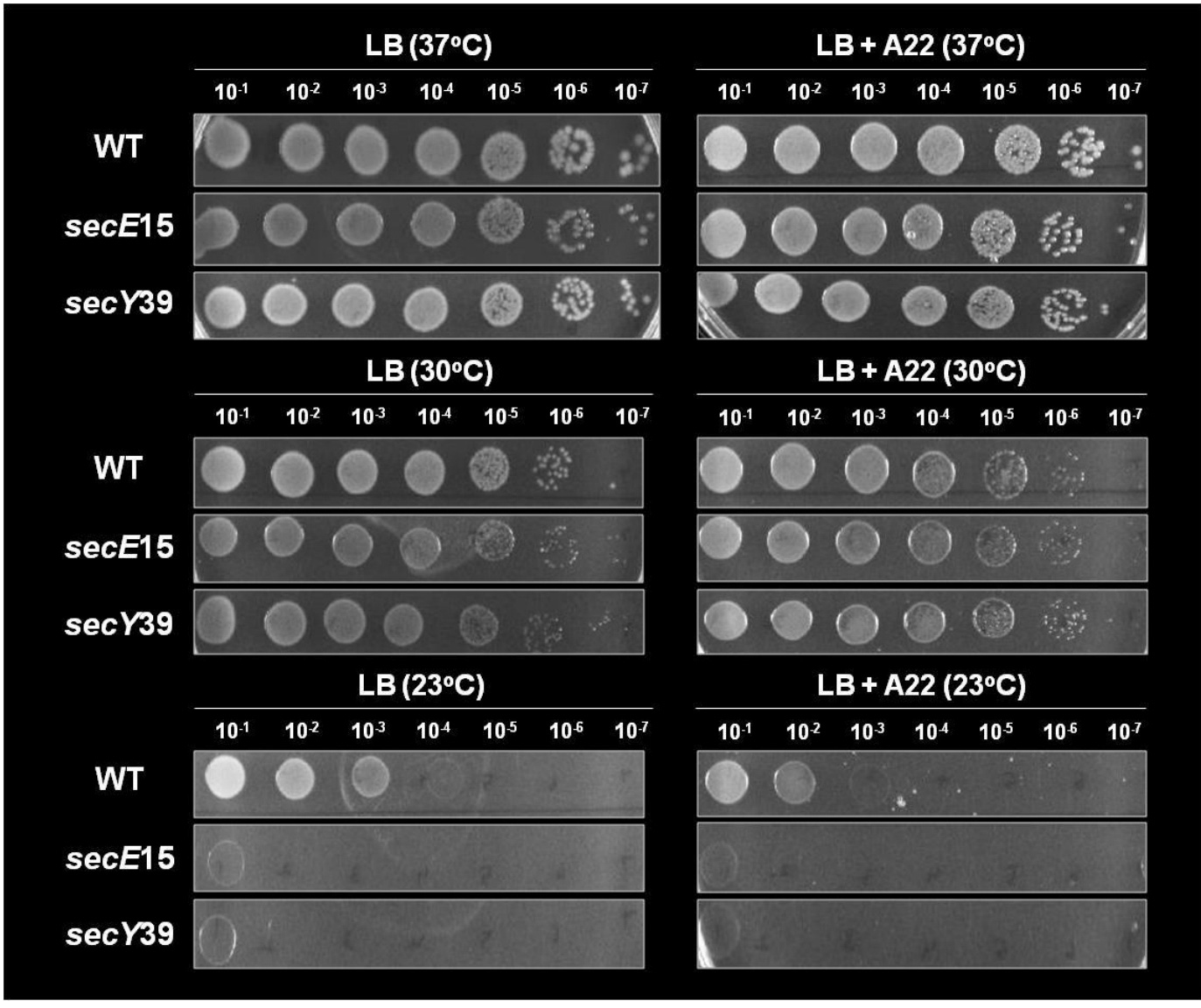

Supplement: S1 Fig — Pictures of wild-type, secE15cs and secY39cs cells spotted after serial dilutions on LB plates with or without sub-inhibitory concentration of A22 (1 μg/ml) and grown for 20 hours at the permissive (37°C), semi-permissive (30°C) or non-permissive (23°C) temperatures. (TIF) [file pgen.1007017.s004.tif]

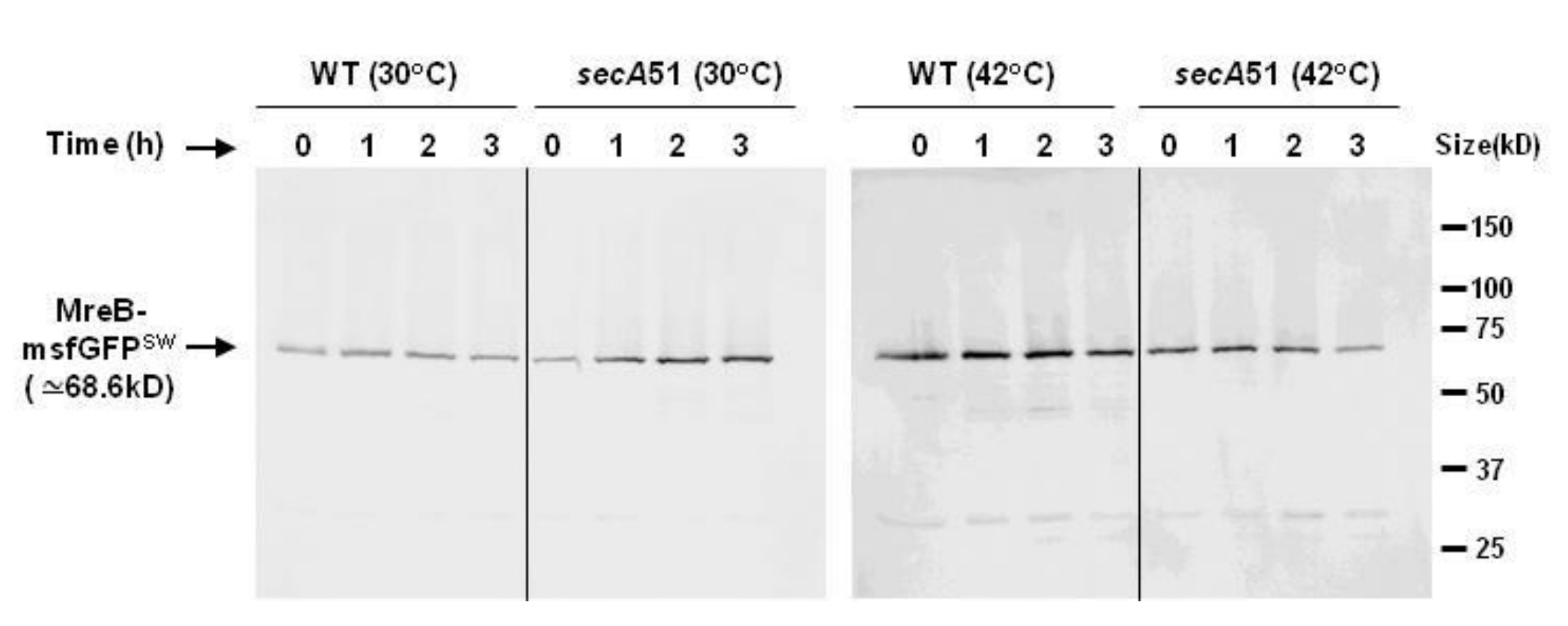

Supplement: S2 Fig — Cells were grown at the permissive (30°C) or restrictive (42°C) temperatures and equal amount of samples were collected at specific time points (0h, 1h, 2h and 3h). Samples were separated on 10% SDS polyacrylamide gel, blotted onto nitrocellulose membrane and detected by α-GFP antibodies. (TIF) [file pgen.1007017.s005.tif]

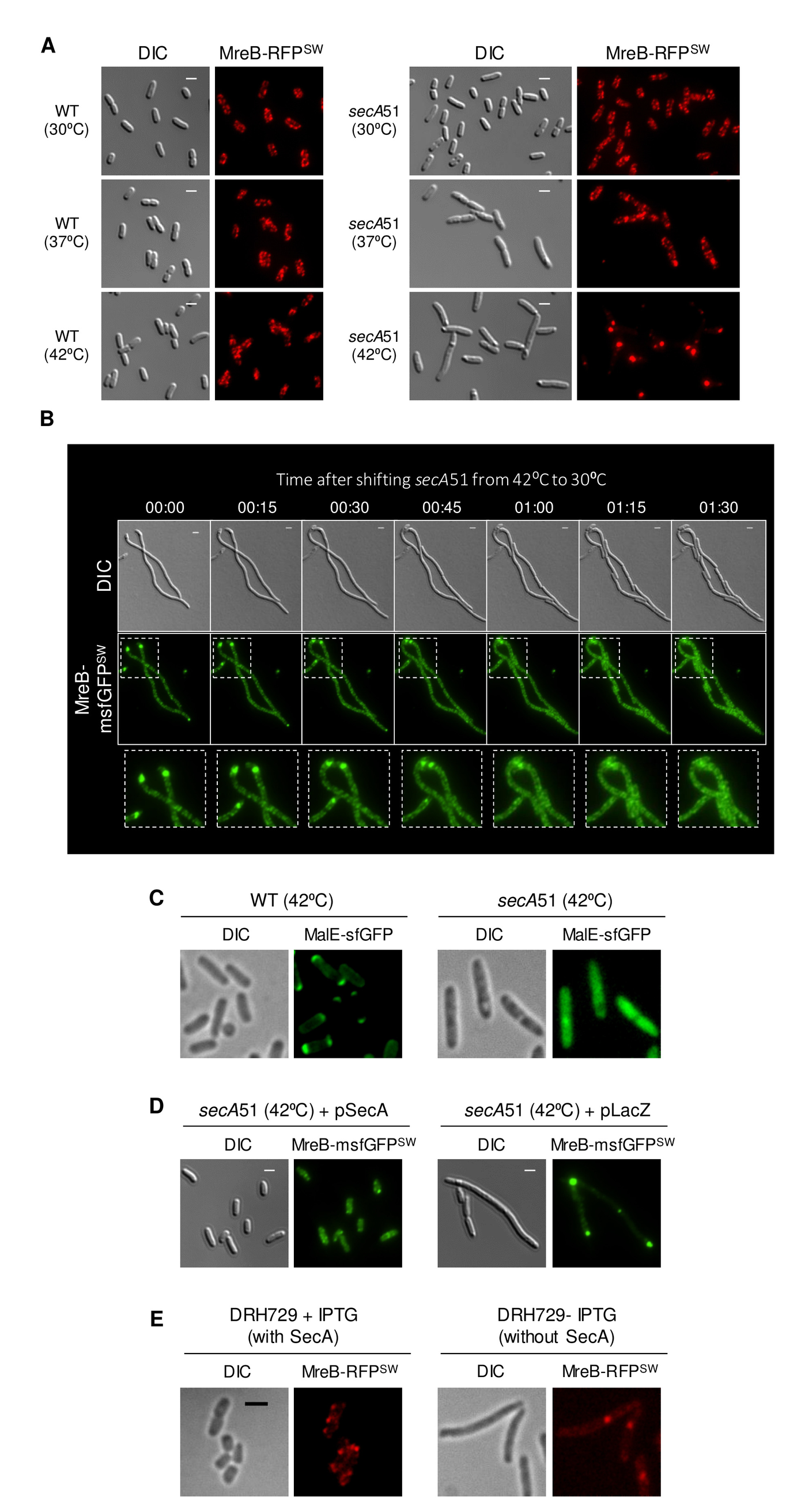

Supplement: S3 Fig — (A) Images of wild-type and secA51 mutant cell, which express MreB-RFPSW, grown at the permissive (30°C), semi-restrictive (37°C), or restrictive (42°C) temperatures. (B) Time-lapse microscopy images of secA51 mutant cells, which express MreB-msfGFPSW, shifted from the non-permissive temperature (42°C) to the permissive temperature (30°C). (C) Images of wild-type and secA51 mutant cell, which express MalE-sfGFP, grown at the non-permissive temperature (42°C). (D) Images of secA51cells, which express MreB-msfGFPSW from the chromosome and wild-type SecA or LacZ from a plasmid, grown at the non-permissive temperature (42°C). (E) Images of IPTG-controlled secA strain (DRH729) expressing MreB-RFPSW in the presence or absence of IPTG. The mCherry and GFP fusion protein were observed by fluorescence microscopy (red and green, respectively), and cells were observed with DIC microscopy (grey). Scale bar corresponds to 2 μm. (TIF) [file pgen.1007017.s006.tif]

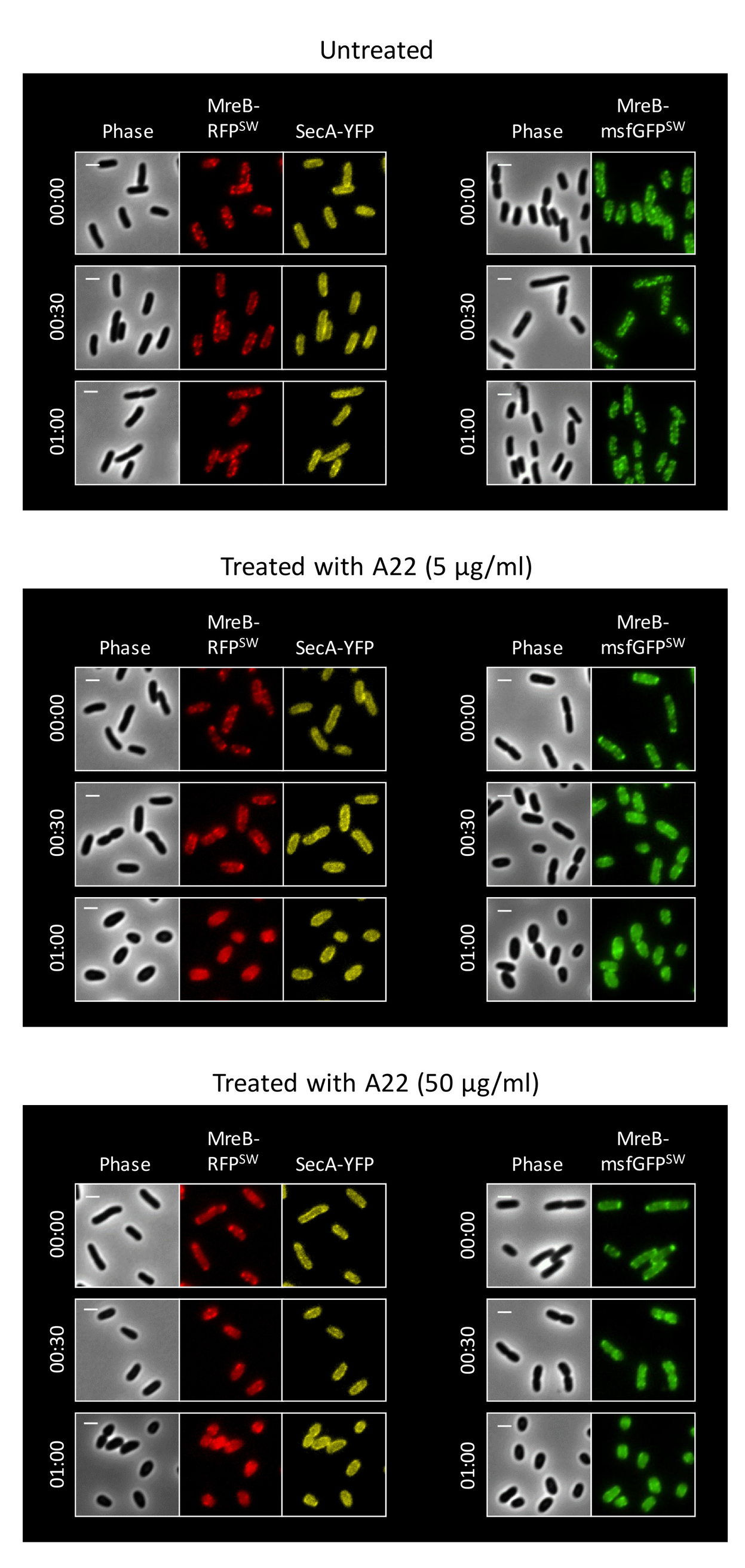

Supplement: S4 Fig — Images of cells expressing MreB-RFPSW and SecA-YFP (together) or MreB-msfGFPSW and untreated (upper panels) or treated with 5 μg/ml of A22 (middle panels) or 50 μg/ml of A22 (lower panels). The mCherry, GFP and YFP fusion proteins were observed by fluorescence microscopy (red, green and yellow, respectively) and cells were observed with phase microscopy (grey). Merges of the YFP and mCherry fluorescence signals are also shown. (TIF) [file pgen.1007017.s007.tif]

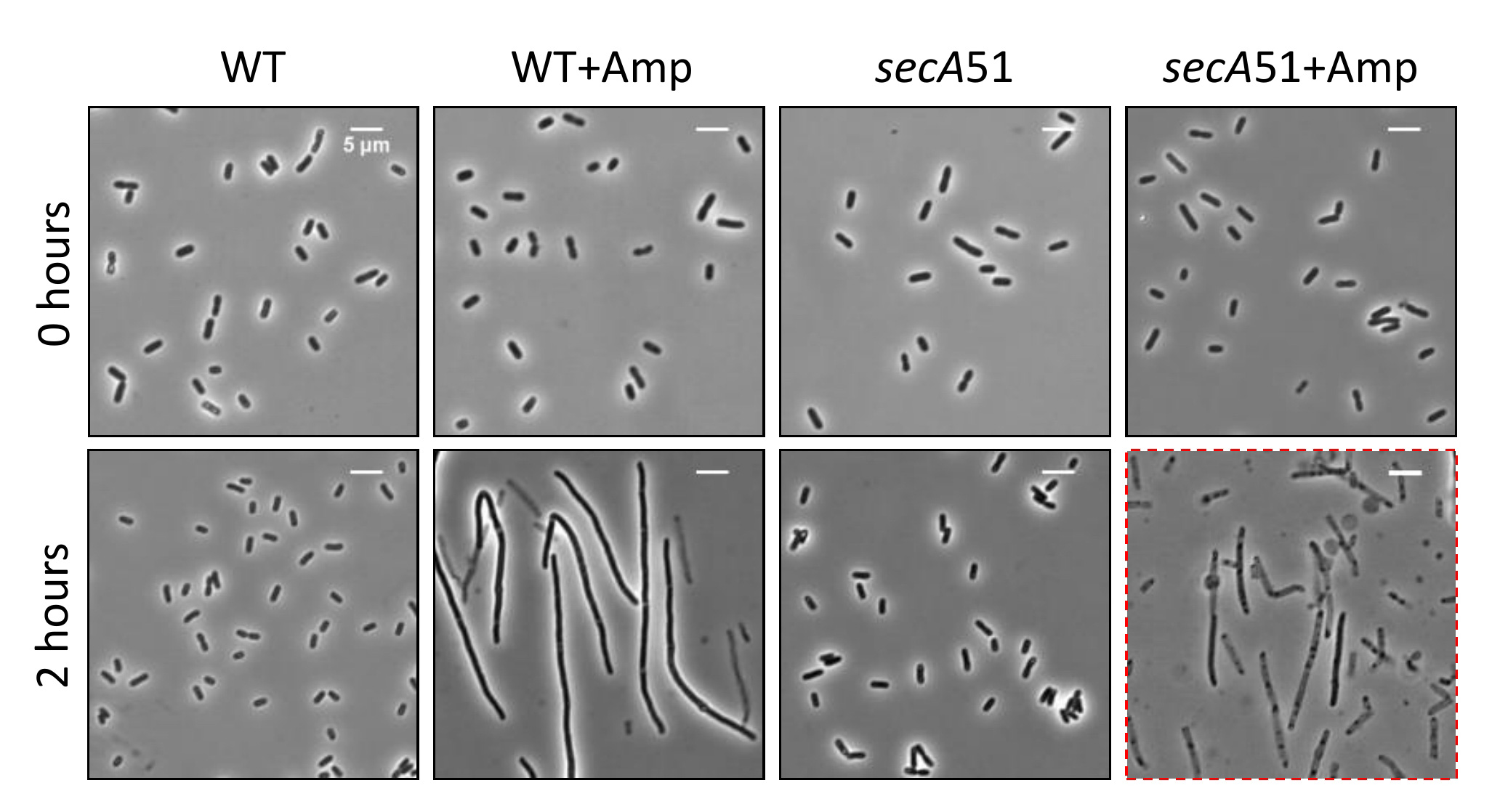

Supplement: S5 Fig — Images of wild-type and secA51 cells grown with or without ampicillin at a semi-restrictive temperature (37°C). Dotted red boxed images indicate cell lysis. Cells were observed with phase microscopy (grey). Scale bar corresponds to 2 μm. (TIF) [file pgen.1007017.s008.tif]

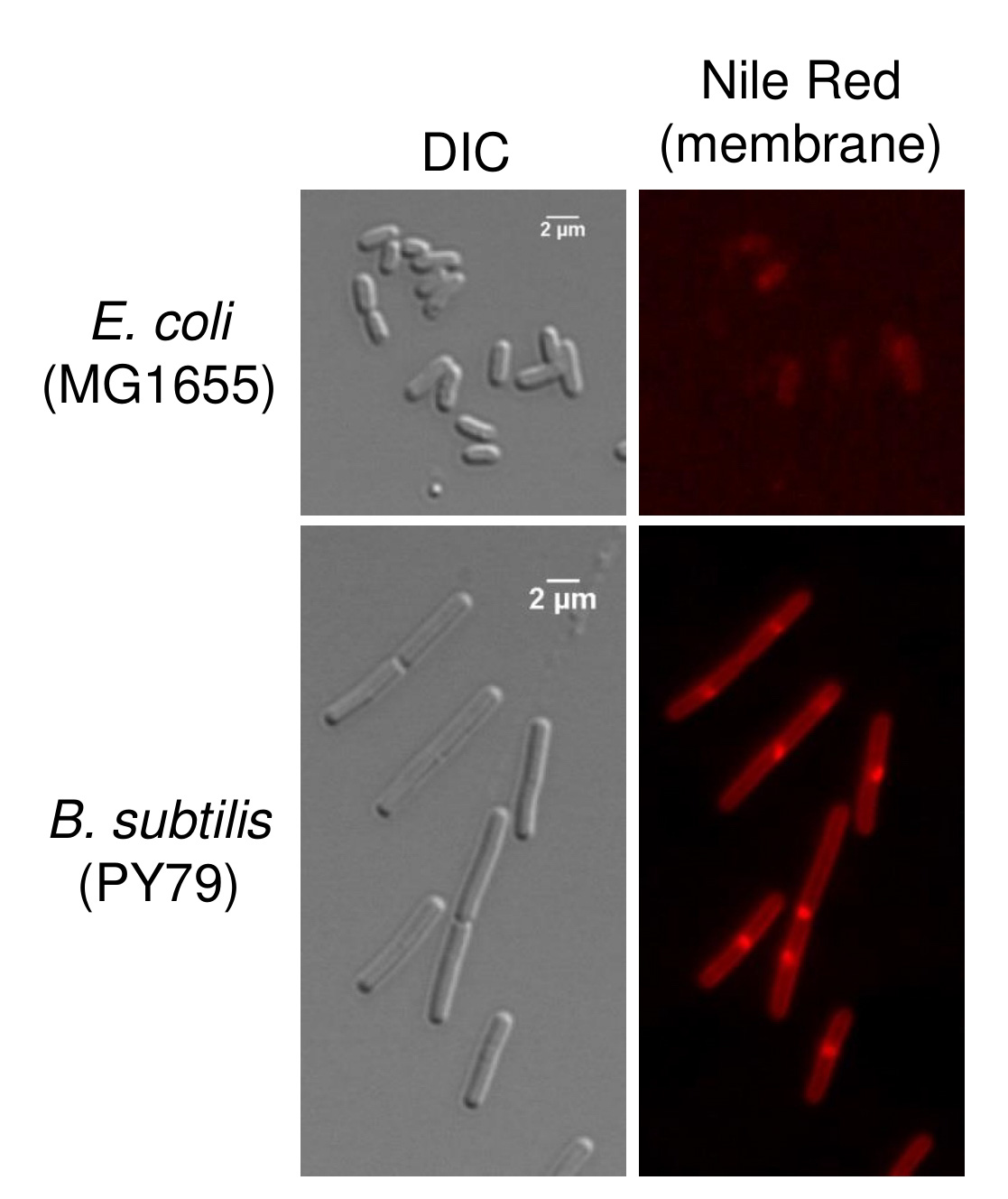

Supplement: S6 Fig — Images of E. coli MG1655 (upper panel) and B. subtilis PY79 cells (lower panel) stained with the fluidity-sensitive dye Nile Red. Staining by Nile Red was observed by fluorescence microscopy (red) and cells were observed with DIC microscopy (grey). Scale bar corresponds to 2 μm. (TIF) [file pgen.1007017.s009.tif]

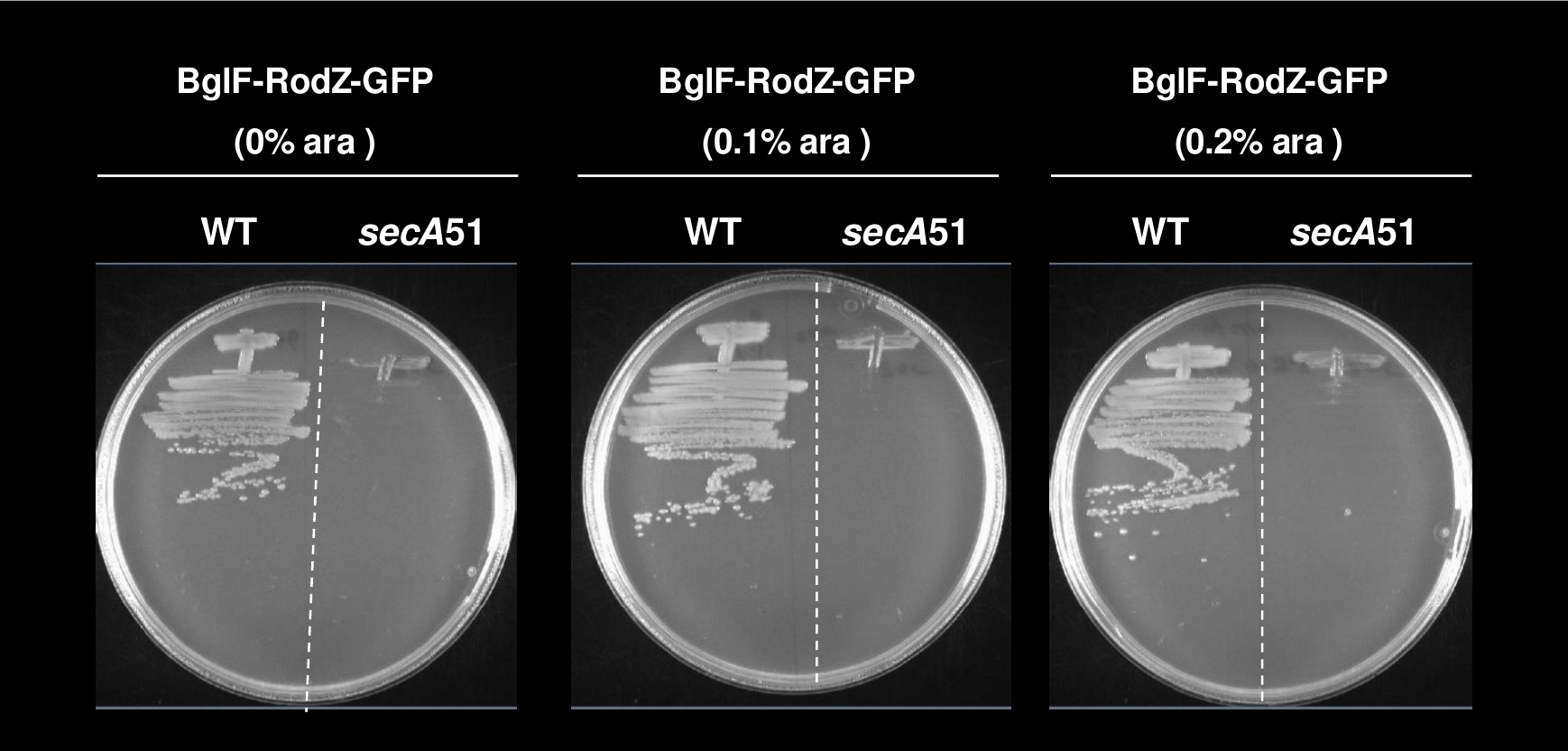

Supplement: S7 Fig — Pictures of wild-type (MG1655) and secA51 cells, containing a plasmid that expresses BglF-RodZ-GFP from the Ara promoter, which were streaked on LB plates and incubated at the non-permissive (42°C) temperature for overnight. The concentration of the arabinose added to each plate are indicated. (TIF) [file pgen.1007017.s010.tif]
