# Supplementary material for: The bacterial Sec system is required for the organization and function of the MreB cytoskeleton
Source: PLoS Genet. 2017 Sep 25;13(9):e1007017. doi: 10.1371/journal.pgen.1007017 (PMC5629013; doi:10.1371/journal.pgen.1007017)
Supplement: S1 Text — (DOCX) [file pgen.1007017.s015.docx]

**Supplemental Experimental Procedures**

**Construction of strains**

SUT101, *secA*51 derivative of MG1655, was constructed by transferring the *secA*51 allele from CWB213 to the chromosome of MG1655 by P1 transduction, using *leuB*::Tn*10* linked marker. Trandcutants were further screened for temperature sensitivity. SUT102 and SUT202, which expresses MreB-RFP^SW^ or MreB-msfGFP^SW^, respectively, in *secA*51 background, were constructed by transduction of the *secA*51 allele linked to *leuB*::Tn*10* from CWB213 to FB76 or NO50, respectively. SUT103 and SUT104 were constructed by evicting the *cat* cassette from FB74 and SUT102, respectively, using FLP recombinase from pCP40. SUT104::∆*rodZ* was constructed by transferring the ∆*yfgA::kan* (*yfgA* is the former name of*rodZ*) from JW2500-1 [4] to the chromosome of SUT104 by P1 transduction. Trandcutants were verified to form round cells. SUT106 and SUT107 were constructed by transduction of *zapA-gfp(::cat)* from HC261 to SUT103 and SUT104, respectively. *zapA-gfp*’frt’was constructed by evicting the *cat* cassette from HC26 using FLP recombinase from pCP40. SUT109, an ∆*mre-678 secA*51 double mutant, was constructed by transferring the *secA*51 allele linked to *leuB*::Tn*10* from CWB213 to PA340-678. SUT110, which expresses MreB-RFP^SW^ and SecA-YFP from the chromosome, was constructed by transduction of *secA-yfp(::cat)* from SX1061 to SUT103. SUT113, which expresses MreB-RFP^SW^ in *secE*15 background, was constructed by transferring *mreB’-mCherry-‘mreB* *yhdE(::cat)* from FB76 to PS266. SUT114 which expresses MreB-msfGFP^SW^ in *secY*39 background, was constructed by transferring *mreB’-msfGfp-‘mreB* *csrD(::neo)* from NO50 to RO257. SUT115, which expresses MreB-RFP^SW^ in an IPTG-controllable SecA strain DRH729, was constructed by transferring *mreB’-mCherry-‘mreB* *yhdE(::cat)* from FB76 to DRH729. *C. crescentus* strains, which express MreB-GFP under the control of xylose promoter, were constructed by transforming strains LS107 and LS416 with the LS3813 plasmid as previously described [11].

**Construction of plasmids**

pGS::N-mGFP, used for construction of N-terminal mGFP fusion proteins, was constructed as following. First, mGFPmut2-encoding sequence, which contains an A206K substitution in the *gfp* gene, was amplified by PCR from pBADGS::EI-mGFP [12] using F-BamHI-mGFP-N and R-SacI-mGFP-N primers and ligating the amplified fragment to BamHI- and SacI-cleaved pQE32-lacI^q^ [13]. R-SacI-mGFP-N primer contains a linker sequence AGCTGCAGC which codes for three alanine residues. pGS::mGFP-RodZ was constructed as follows: *rodZ* ORF sequence was amplified from *E. coli* MG1655 genomic DNA using F-SacI-rodZ and R-XmaI-rodZ primers. The amplified fragment was inserted into SacI- and XmaI-cleaved pGS::N-mGFP. pBAD-RodZ-GFP was constructed as follows: *rodZ* was amplified without the stop codon using F-NheI-rbs-notI-rodZ and R-sacI-rodZ-TAA- primers from the chromosome of MG1655 wild-type *E. coli*. The forward primer contains RBS site, AAGAAGGAGA, which is the RBS site of pET15b expression vector. The amplified product was inserted into NheI- and SacI-cleaved pBADANSHPr-GFP, thus replacing *ptsH*, which was inserted between these sites in pBADANSHPr-GFP. pBAD-BglF-RodZ-GFP was constructed as follows: *bglF* was amplified without the stop codon using F-NheI-rbs-bglF and R-NotI-bglF-full(TAA-) primers. Forward primer amplified *bglF* 18 bp before the ATG, thus including the native RBS of *bglF*. The amplified product was digested with NheI and NotI and inserted into NheI-NotI cleaved pBAD-RodZ-GFP.

**Growth conditions used for snapshot imaging**

For snap-shot imaging, samples are spotted on 1% M9 glycerol agarose pads with uncoated cover-slips or on poly-lysine coated coverslips before they were visualized in the microscope. MreB-RFP^SW^, MreB-msfGFP^SW^, ZapA-GFP and SecA-YFP were all expressed from the native chromosomal locus. To visualize MreB-msfGFP^SW^ in WT and SecA-defective cells, strains NO50 and SUT202 were used, respectively. To visualize MreB-RFP^SW^ in WT and SecA-defective cells, strains FB76 and SUT102 were used, respectively. Overnight cultures, grown in M9 glycerol at 30^o^C and supplemented with appropriate antibiotic, were sub-cultured by 1:100 dilution in fresh M9 glycerol medium. Cells were allowed to grow at the permissive temperature (30^o^C) till they reached OD_600_ of 0.12 to 0.15. Cultures were either shifted to the restrictive temperature (42^o^C) or continued to grow at the permissive temperature for additional 3 hours. Unless indicated, the same growth conditions were used for SecA depletion in all other experiments. For complementation experiments, wild-type SecA or LacZ, expressed from pCA24N-*secA* or pCA24N*-lacZ* respectively, were expressed without the addition of IPTG. For over-expression of wild-type SecA or LacZ in strains expressing MreB-msfGFP^SW^ or ZapA-GFP from the chromosome, overnight cultures were sub-cultured by 1:500 dilution in fresh M9 glycerol medium supplmented with 0.1 mM IPTG and cells were grown at 30^o^C for 8 hours with slow shaking (100 rpm). To express MalE-sfGFP from pLac::MalE-sfGFP, IPTG (0.1 mM) was added during the last three hours of growth at the restrictive temperature. Expression of mGFP-RodZ from the pGS::mGFP-RodZ was not induced. Expression of RodZ-GFP from pBAD-RodZ-GFP was induced with 0.1% arabinose during the last one hour of the total three hours of growth at the restrictive temperature. For experiments involving partial translation suppression, chloramphenicol (1 µg/ml) was added during the last three hours of growth when cultures were either shifted or not shifted to 42^o^C. Expression of BglF-RodZ-GFP from pBAD-BglF-RodZ-GFP was induced with 0.1% arabinose when cultures were shifted to grow at at the restrictive temperature (last 3 hours at 42^o^C). To visualize MreB-RFP^SW^ in *secE*15 cells or MreB-msfGFP^SW^ in *secY*39 cells, overnight cultures were sub-cultured 1:1000 in fresh L-medium [(1% bactotryptone (Difco), 0.5% yeast extract (Difco), and 0.5% NaCl (pH 7.4)] and grown at the permissive temperature (37^o^C), semi-permissive (30^o^C) or at the restrictive temperature (23^o^C) till they reached OD_600_ of 0.4 to 0.6. Both *secE*15 cells and *secY*39 cells took nearly 15-20 hours to reach OD_600_ of 0.4 to 0.6 at 23^o^C. To visualize MreB-RFP^SW^ in SecA-depleted cells, overnight cultures of SUT115 cells grown in M9 glucose medium with 20 µM IPTG were sub-cultured 1:100 in fresh M9 glucose medium with or without 20 µM IPTG. Cells were grown at 30^o^C for 5 to 8 hours for SecA depletion. For imaging of MreB-RFP^SW^ together with SecA-YFP, under conditions of MreB disruption, strain SUT110, which expresses both proteins from their native chromosomal loci, was grown in LB at 30^o^C and supplemented with appropriate antibiotic; cells were sub-cultured 1:100 in fresh LB medium and grown at 30^o^C till they reached OD_600_ of 0.25 to 0.3; at this point, A22 (5 μg/ml or 50 μg/ml) was added or not. A22-treated and untreated samples, collected at time points 0 mins, 30 mins and 1 hour, were spotted on 1% TB-agarose pads with respective concentration of A22, before they were visualized in the microscope (TB composition: 10% Tryptone and 5% NaCl). Similar conditions were used to dirupt MreB in NO50 cells. To visualize MreB-GFP in WT and SecA-defective *C. crescentus* cells, strains LS107::MreB and LS416::MreB were used, respectively. Overnight cultures, grown in PYE medium at 30^o^C and supplemented with kan (5 µg/ml), were sub-cultured to an initial OD_600_ of 0.04 in fresh PYE medium. Cells were allowed to grow at the permissive temperature (30^o^C) till they reached OD_600_ of 0.12 to 0.15. At this point, the inducer for GFP-MreB expression, xylose (0.1%), was added and the cultures were either shifted to the restrictive temperature (37^o^C) or continued to grow at the permissive temperature (30^o^C) for additional 3 hours before imaging.

**Growth conditions used for time-lapse imaging**

Ariekacells^R^ coverslip cell chamber (SC15012) was used for time-lapse imaging. For time-lapse imaging, samples are spotted on 1% agarose pads with M9-glycerol (0.2%) medium, before they were visualized in the microscope. For time-lapse imaging of MreB-msfGFP^SW^ in WT and SecA-defective cells, NO50 and SUT202 were used. For time-lapse imaging of MreB-RFP^SW^ in WT and SecA-defective cells, FB76 and SUT102 were used. Overnight cultures were sub-cultured 1:100 in fresh M9 glycerol medium. Cells were allowed to grow at the permissive temperature (30^o^C) till they reached OD_600_ of 0.12 to 0.15. Culture samples were spotted onto pre-set 1% M9 glycerol pads, which had been pre-equilibrated to 42^o^C and imaged immediately for time-lapse microscopy. The same conditions were used for time-lapse microscopy of SUT106 and SUT107 strains, which expresses MreB-RFP^SW^ together with ZapA-GFP, in WT and SecA-defective cells, respectively. To calculate the percentage of cells with MreB-Z-ring colocalization (Fig. 6C), time-lpase microscopy for SUT106 and SUT107 strains was performed for 2 hours under conditions of SecA-inactivation. In order to visualize the reversal of localization of MreB-msfGFP^SW^ assemblies, SUT202 cells, which expresses MreB-msfGFP^SW^ in *secA*51 background, were initially grown under SecA depletion conditions that promoted its aggregation. Culture samples were then spotted onto pre-set 1% M9 glycerol pads and imaged for 1 hour in time-lapse microscopy at 30^o^C.

**Sample preparation and TEM analysis**

For TEM analysis, overnight cultures of MG1655, SUT102 and SUT109 were grown in M9 glycerol medium at 30^o^C and sub-cultured 1:100 in fresh M9 glycerol medium. Cells were allowed to grow at the permissive temperature (30^o^C) till they reached OD_600_ of 0.12 to 0.15. Cultures were shifted to the restrictive temperature (42^o^C) for additional 3 hours. Cells were harvested, washed twice in PBS (Phosphate Buffered Saline pH 7.4) and then fixed in 2% paraformaldehyde and 2.5% Glutaraldeyde in 0.1M Cacodylate buffer (pH 7.4) for 2.5 hours at room temperature and then moved to 40^o^C for another 16 hours. The bacteria were then rinsed 4 times, 10 minutes each, in cacodylate buffer and post fixed and stained with 1% osmium tetroxide, 1.5% potassium ferricyanide in 0.1M cacodylate buffer for 1 hour. Each step was followed by centrifugation at 1,300g for 4 minutes. Bacteria were then washed 4 times in cacodylate buffer followed by dehydration in increasing concentrations of ethanol consisting of 30%, 50%, 70%, 80%, 90%, 95%, for 10 minutes each step followed by 100% anhydrous ethanol 3 times, 20 minutes each, and propylene oxide 2 times, 10 minutes each. Following dehydration, the cells were infiltrated with increasing concentrations of Agar 100 resin in propylene oxide, consisting of 25, 50, 75, and 100% resin for 16 hours each step. The bacteria were then embedded in fresh resin and let polymerize in an oven at 600^o^C for 48 hours. Embedded bacteria in blocks were sectioned with a diamond knife on an LKB 3 microtome and ultrathin sections (80 nm) were collected onto 200 Mesh, thin bar copper grids. The sections on grids were sequentially stained with Uranyl acetate and Lead citrate for 10 minutes each and viewed with Tecnai 12 TEM 100kV (Phillips, Eindhoven, the Netherlands) equipped with MegaView II CCD camera and Analysis® version 3.0 software (SoftImaging System GmbH, Münstar, Germany).

**Cell wall labeling using HADA**

MG1655, SUT101 (*secA*51), NO50 (wild-type MreB-msfGFP^SW^) and SUT202 (*secA*51 MreB-msfGFP^SW^) were grown at the *secA*51 restrictive temperature as described above. The cell wall was stained with fluorescent HCC-amino-D-alanine (HADA). Cells were washed with fresh M9 glycerol medium, normalized to 0.5 OD and resuspended in 0.5 ml of fresh M9 glycerol medium, which contained the indicated concentrations of HADA. Cultures were incubated at 42^o^C for 30 minutes with shaking. Unstained or HADA-stained cells were washed three times and resuspended in 1X PBS before they were imaged by fluorescence microscopy. For quantification of HADA fluorescence in wild-type and s*ecA*51 cells (Fig. 4B), 10 µM, 100 µM and 200 µM HADA were used.

For visualization of cell wall (Fig. 4A), cells were stained with 200 µM HADA, as described above, washed three times and resuspended in 1X PBS before they were imaged by fluorescence microscopy.

For time-lapse pulse-chase microscopy (Fig. 4C), cells were stained with 1 mM HADA, as described above, washed four times and resuspended in fresh M9 glycerol without HADA. Samples were then spotted onto pre-set 1% M9 glycerol pads without HADA and imaged for 30 minute in time-lapse microscopy at 42^o^C.

I**mage analysis**

*Calculating the fraction of mislocalized MreB in secA51 cells:* Using NIS-Elements Advanced Research (AR), regions of interest (ROI) were drawn over to the phase contrast images of 50 cells from two different experiments. From these ROI, the sum intensity of MreB-msfGFP^SW^ fluorescence (after substracting the background) from the 50 individual cells were obtained (total sum intensity). Next, for each cell, we drew a new ROI over the mislocalized MreB foci and obtained the sum intensity of the mislocalized MreB-msfGFP^SW^ foci for the individual cells. The ratio of mislocalized MreB in individual cells is obtained by dividing the total sum intensity of individual cells by their respective sum intensity of mislocalized MreB-msfGFP^SW^ foci.

*Measuring the average MreB-msfGFP^SW^ fluorescence and variance of MreB-msfGFP^SW^ fluorescence in wild-type and secA51 cells:* Using NIS-Elements Advanced Research (AR), regions of interest (ROI) were drawn over to the phase contrast images of approximately 100 wild-type and *secA*51 cells, expressing MreB-msfGFP^SW^, from two different experiments. From each ROI, the values of mean intensity and standard deviation from the GFP channel (after substracting the background) for each cell were obtained. For calculating the average MreB-msfGFP^SW^ fluorescence in wild-type and *secA*51 cells, the mean intensity values of the GFP signal were analyzed using GraphPad Prism and presented as a scatter plot (Fig. 2C, left). For calculating the variance of MreB-msfGFP^SW^ fluorescence, standard deviation values from the GFP channel were obtained. Variance among the standard deviation values were calculated from Excel using the variance function. The values obtained were exported and analysed by GraphPad prism and presented as a scatter plot (Fig. 2C, right).

*Measuring the average HADA fluorescence of wild-type and secA51 cells:* Using NIS-Elements AR module, ROI were drawn over the phase contrast images of HADA-labelled wild-type and *secA*51 cells. Of note, ROI were drawn slightly beyond the limit of the phase constrast images to ensure that the HADA signal, which is present at the edge of the cells, are located within the ROI. After substracting the background, the HADA ROI mean intensity value were obtained. The data was analyzed and presented as a bar graph using GraphPad prism (Fig. 4B).

**Antibiotic lysis assay**

Antibiotic lysis assay was performed as descrbied previously [14] with minor modifications. Overnight cultures of MG1655 and SUT101 were grown in LB medium at 30^o^C and sub-cultured 1:100 in fresh LB medium. Cells were allowed to grow at the permissive temperature (30^o^C) till they reached OD_600_ of 0.6. Cultures were diluted 1:3 into fresh LB medium with or without ampicillin (5 μg/ml), cefotaxime (1 μg/ml) or rifampicin (5 μg/ml), aliquoted in 96-well titer plates and grown at 37°C, which is a semi-restrictive temperature for Sec-depletion, for 5 hours in Spark multimode microplate reader. The OD_600_ of each culture was measured every 15 min. For direct observation of ampicillin-induced cell lysis, aliquotes of ampicillin-treated or untreated cultures at time points 0 and 2 hours were collected and imaged by phase contrast microscopy.

**Western blotting**

Equal amount of samples were collected, washed and their proteins were separated on 10% SDS–polyacrylamide gels. Gels were subjected to Western blot analysis as described previously [13]. α-GFP antibodies (Abcam) were used for detection of MreB-msfGFP^SW^.

**Supplemental References**

1. Bowers CW, Lau F, Silhavy TJ (2003) Secretion of LamB-LacZ by the signal recognition particle pathway of Escherichia coli. Journal of bacteriology 185: 5697-5705.

2. Ursell TS, Nguyen J, Monds RD, Colavin A, Billings G, et al. (2014) Rod-like bacterial shape is maintained by feedback between cell curvature and cytoskeletal localization. Proceedings of the National Academy of Sciences 111: E1025-E1034.

3. Bendezu FO, Hale CA, Bernhardt TG, de Boer PAJ (2009) RodZ (YfgA) is required for proper assembly of the MreB actin cytoskeleton and cell shape in E. coli. The EMBO journal 28: 193-204.

4. Baba T, Ara T, Hasegawa M, Takai Y, Okumura Y, et al. (2006) Construction of Escherichia coli K-12 in-frame, single-gene knockout mutants: the Keio collection. Molecular systems biology 2.

5. Peters NT, Dinh T, Bernhardt TG (2011) A fail-safe mechanism in the septal ring assembly pathway generated by the sequential recruitment of cell separation amidases and their activators. Journal of bacteriology 193: 4973-4983.

6. Wachi M, Doi M, Okada Y, Matsuhashi M (1989) New mre genes mreC and mreD, responsible for formation of the rod shape of Escherichia coli cells. Journal of bacteriology 171: 6511-6516.

7. Taniguchi Y, Choi PJ, Li G-W, Chen H, Babu M, et al. (2010) Quantifying E. coli proteome and transcriptome with single-molecule sensitivity in single cells. Science 329: 533-538.

8. Kang PJ, Shapiro L (1994) Cell cycle arrest of a Caulobacter crescentus secA mutant. Journal of bacteriology 176: 4958-4965.

9. Dinh T, Bernhardt TG (2011) Using superfolder green fluorescent protein for periplasmic protein localization studies. Journal of bacteriology 193: 4984-4987.

10. Kitagawa M, Ara T, Arifuzzaman M, Ioka-Nakamichi T, Inamoto E, et al. (2006) Complete set of ORF clones of Escherichia coli ASKA library (a complete set of E. coli K-12 ORF archive): unique resources for biological research. DNA research 12: 291-299.

11. Gitai Z, Dye N, Shapiro L (2004) An actin-like gene can determine cell polarity in bacteria. Proceedings of the National Academy of Sciences of the United States of America 101: 8643-8648.

12. Govindarajan S, Elisha Y, Nevo-Dinur K, Amster-Choder O (2013) The general phosphotransferase system proteins localize to sites of strong negative curvature in bacterial cells. MBio 4: e00443-00413.

13. Lopian L, Elisha Y, Nussbaum-Shochat A, Amster-Choder O (2010) Spatial and temporal organization of the E. coli PTS components. The EMBO journal 29: 3630-3645.

14. Uehara T, Dinh T, Bernhardt TG (2009) LytM-domain factors are required for daughter cell separation and rapid ampicillin-induced lysis in Escherichia coli. Journal of bacteriology 191: 5094-5107.
